# The accessory protein ORF3a hijacks the CLCC1 chloride channel to disrupt ER homeostasis in betacoronavirus pathogenesis

**DOI:** 10.1038/s41421-026-00918-0

**Published:** 2026-09-15

**Authors:** Liang Guo, Baoying Huang, Hanzhi Yu, Yi Xu, Lei Wei, Jijie Zheng, Yuanzhe Li, Di Wu, Peng Zhao, Changcheng Wu, Wenjie Tan, Yichang Jia

**Affiliations:** 1 State Key Laboratory of Complex, Severe, and Rare Diseases, Tsinghua Medicine, Tsinghua University, Beijing, China; 2 Aging and Regeneration Center, School of Basic Medical Sciences, Tsinghua Medicine, Tsinghua University, Beijing, China; 3 Tsinghua-PKU Joint Center for Life Sciences, Beijing, China; 4 IDG/McGovern Institute for Brain Research at Tsinghua, Beijing, China; 5 Tsinghua Laboratory of Brain and Intelligence, Beijing, China; 6 National Key Laboratory of Intelligent Tracking and Forecasting for Infectious Diseases, NHC Key Laboratory of Biosafety, National Institute for Viral Disease Control and Prevention, Chinese Center for Disease Control and Prevention, Beijing, China; 7 School of Pharmaceutical Sciences, Tsinghua University, Beijing, China; 8 School of Life Sciences, Tsinghua University, Beijing, China; 9 MOE Key Laboratory of Bioinformatics and Bioinformatics Division, BNRIST, Department of Automation, Tsinghua University, Beijing, China; 10 SXMU-Tsinghua Collaborative Innovation Center for Frontier Medicine, Shanxi Medical University, Taiyuan, Shanxi, China; 11 Present Address: Department of Immunology, School of Basic Medical Sciences, Harbin Medical University, Harbin, Heilongjiang, China

**Keywords:** Endoplasmic reticulum, Autophagy, Mechanisms of disease

## Abstract

Beta-coronavirus infection disrupts endoplasmic reticulum (ER) homeostasis; however, the mechanisms by which viral proteins manipulate ER-resident factors remain unclear. Here, we report that the SARS-CoV-2 accessory protein ORF3a binds to the ER chloride channel CLCC1, thereby impairing ER ion homeostasis, activating the unfolded protein response, and inducing endomembrane remodeling. Using newly developed ratiometric reporters, we showed that this interaction exacerbated the basal levels of ER-phagy and nucleophagy. Importantly, CLCC1 counteracted ORF3a by sequestering it within the ER, attenuating its toxicity and suppressing viral replication, which established CLCC1 as a host restriction factor. We further elucidated a spatiotemporal mechanism: during early infection, ORF3a accumulates in the ER, co-assembles with CLCC1 into puncta, and disrupts ER homeostasis; at the later stage, ORF3a overload enables its escape from CLCC1-mediated ER retention and subsequent translocation to lysosomes, facilitating viral egress. This ER-centric function is conserved across diverse beta-coronaviruses, including SARS-CoV-1 and bat- or pangolin-derived strains, revealing a unified pathogenic strategy. Furthermore, in mouse brains, the ORF3a-CLCC1 axis recapitulates key neuropathological features, including ER stress, autophagic dysfunction, neuronal death, and neuroinflammation, providing a mechanistic link to COVID-19-associated neurological symptoms. Our findings identify CLCC1 as an essential host defense protein and establish a conceptual framework to guide future research on antiviral responses at the organelle level and related disease mechanisms.

## Introduction

Severe acute respiratory syndrome coronavirus 2 (SARS-CoV-2) caused the COVID-19 pandemic, which presented a global health crisis. COVID-19 primarily affects the respiratory system but can also manifest neurological symptoms, including headaches, dizziness, loss of taste or smell (anosmia or ageusia), cognitive impairment, strokes, seizures, and neuromuscular symptoms^1–3^. However, the underlying pathogenic mechanisms remain not fully understood. Although COVID-19 is no longer a global health emergency, it remains a public health threat (https://news.un.org/en/story/2024/08/1152866). It is therefore imperative to explore the underlying mechanisms fully to aid in the development and preparation of targeted therapies for future surges of viral infections.

SARS-CoV-2, a beta-coronavirus in the *Orthocoronavirinae* subfamily, harbors single-stranded viral genomic RNA (gRNA, ~30 kb)^4^. After SARS-CoV-2 infection, the gRNA is released from the viral particle and translated and processed into 16 non-structural proteins (NSPs), which assemble into replication–transcription complexes (RTCs). The resulting RTCs are responsible for the replication of gRNAs and transcription of subgenomic mRNAs, the latter of which encode structural spike (S), membrane (M), envelope (E), and nucleocapsid (N) proteins, as well as several accessory proteins^5,6^.

SARS-CoV-2 accessory proteins, including ORF3a, have been implicated in determining viral pathogenesis and modulating host immune responses^7,8^. ORF3a is the largest SARS-CoV-2 accessory protein, spanning 275 residues with an N-terminal signal peptide (SP), three transmembrane domains (TMs), and a cytosolic loop featuring beta-sheet structures at its C-terminus^9,10^. ORF3a may function as a viroporin that mediates K^+^ or non-selective cation currents, but the channel activity of ORF3a remains controversial^9–11^. ORF3a is involved in many steps of the viral life cycle, including viral entry and release^8,12,13^. Moreover, ORF3a interacts with host factors to induce innate immune responses and pro-inflammatory reactions^8^. Autophagy is a crucial cellular process for the antiviral response to capture viruses for lysosomal clearance^14^. Among the 29 viral proteins, ORF3a strongly inhibits autophagic flux in host cells^15^. Mechanistically, SARS-CoV-2 ORF3a interacts with VPS39 to inhibit the binding of the homotypic fusion and vacuole protein sorting (HOPS) complex to Rab7 and/or the soluble N-ethylmaleimide-sensitive factor attachment protein receptor (SNARE) protein STX17 to prevent autolysosome formation^12,15,16^. In addition, ORF3a activates the NLRP3 inflammasome, leading to a cytokine storm and causing cell death, contributing to the clinical symptoms of COVID-19^17–20^. Moreover, an ORF3a-specific antibody has been detected in SARS-CoV-2-infected patients^21^, and deletion of ORF3a reduced viral pathogenesis in a K18 human ACE2 (hACE2) transgenic mouse model^22^, indicating its potential clinical value as a target for therapeutic interventions.

NSPs also form various membranous structures, such as perinuclear double-membrane vesicles (DMVs), which are derived mainly from the endoplasmic reticulum (ER) and protect viral RNA under replication and transcription^23–26^. Concurrently, these structural proteins are translated on the ER and then translocated to the ER-to-Golgi intermediate compartment (ERGIC) to form virions (viral particles) by interacting with newly synthesized and encapsidated viral gRNAs^27^. Finally, the virus egresses via lysosomal exocytosis^13,28^. Thus, ER morphology and function are critical for the pathogenicity and transmissibility of SARS-CoV-2.

Current antiviral strategies focus mainly on virus neutralization (vaccine development), protease inhibitors that prevent virus entry and polyprotein processing, viral RNA-dependent RNA polymerase inhibitors that block RNA synthesis, and viral maturation inhibitors that disrupt assembly^4,29^. The ER is not only the factory for viral structural protein synthesis but also the membrane resource of DMVs and the ERGIC^27,30^. However, the potential of ER-resident host factors in combating SARS-CoV-2 remains underexplored. Analysis of the interactome of ORF3a via different biochemical approaches revealed chloride channel CLIC-like 1 (CLCC1) as an interaction partner of ORF3a^31–34^. CLCC1 is an ER-resident chloride channel whose activity is required for the maintenance of ER ionic homeostasis and ER morphology^35^. However, the biological consequences of the interaction between ORF3a and CLCC1 are largely unknown.

Here, we examined the interaction between SARS-CoV-2 ORF3a and CLCC1, revealing that this interaction disrupts ER ion homeostasis, triggers unfolded protein response (UPR), and induces ER-phagy and nucleophagy. Our findings demonstrate that ORF3a induces the formation of endogenous CLCC1 puncta and that overexpression of CLCC1 mitigates ORF3a-associated toxicity and inhibits virus propagation by sequestering ORF3a in the ER. Moreover, our study highlights the conserved functions of ORF3a across beta-coronaviruses in influencing ER function. In addition, SARS-CoV-2 ORF3a exhibits pathologies related to ER functions and morphology in the brains of mouse models, providing novel insights into the neurological symptoms and long-term effects observed in COVID-19 patients, which are caused by the disruption of normal CLCC1 functions in the ER.

## Results

### SARS-CoV-2 ORF3a interacts with CLCC1, impairs ER ion homeostasis, and triggers the UPR

**Fig. 1:**
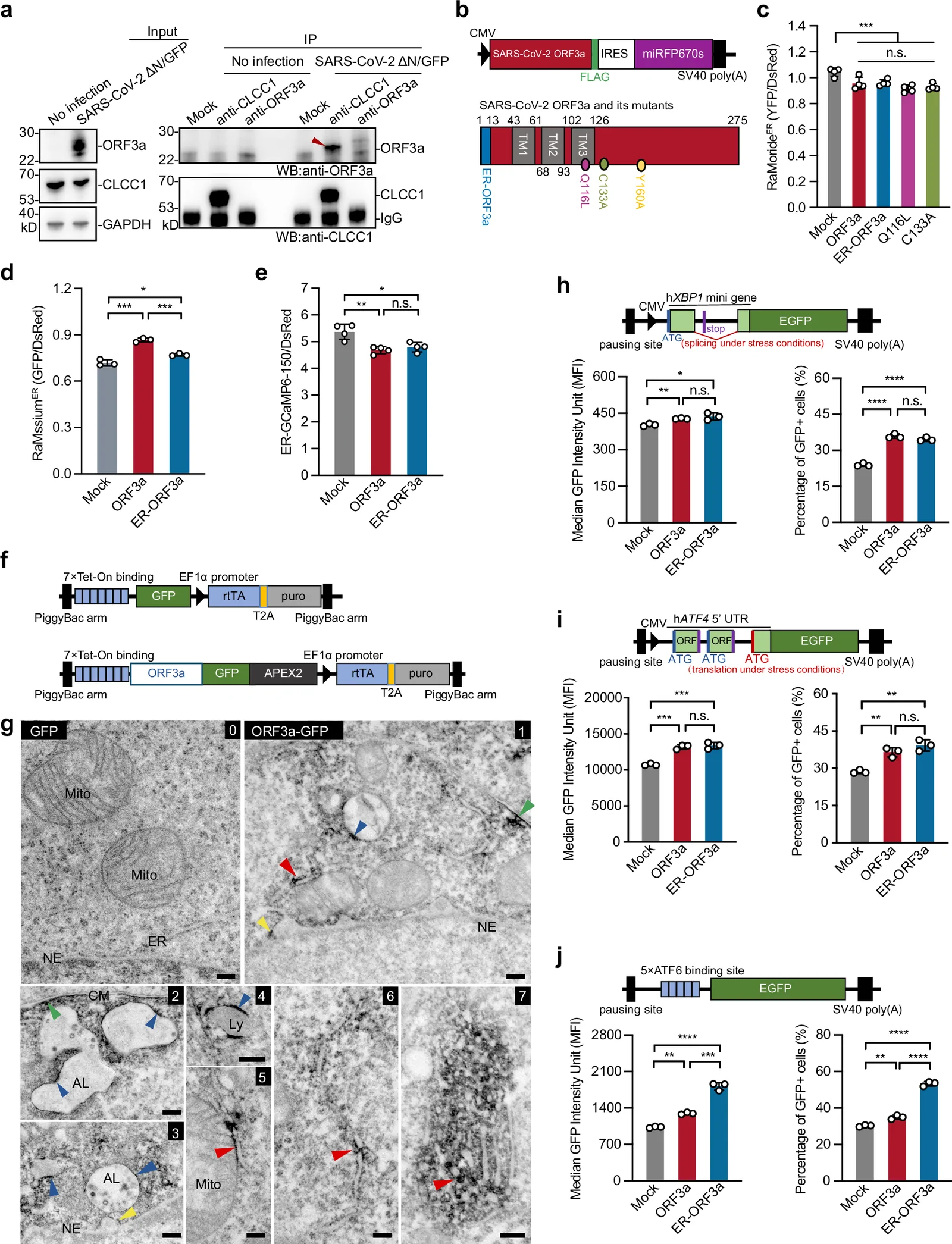
SARS-CoV-2 ORF3a interacts with CLCC1 to impair ER ion homeostasis and trigger the UPR. **a** Western blot analysis of endogenous CLCC1–ORF3a interactions. The cell lysate of Caco-2 cells expressing the N protein and infected with trVLPs was lysed for subsequent co-IP. Endogenous proteins were immunoprecipitated using anti-ORF3a or anti-CLCC1 antibodies. Controls: No infection (Caco-2 cells expressing N protein alone), Mock (rabbit IgG isotype control for IP). GAPDH served as a loading control. **b** Illustration of the constructs of SARS-CoV-2 ORF3a and its mutants. The expression of miRFP670s was controlled by internal ribosome entry sites (IRESs) and was used to monitor the expression of SARS-CoV-2 ORF3a by a FACS assay. ER-ORF3a replaced the 13 N-terminal residues of SARS-CoV-2 ORF3a with the endoplasmic reticulum-targeting sequence of calreticulin. Q116L impairs the cation channel activity of SARS-CoV-2 ORF3a^9^. C133A disrupts the homodimer formation of ORF3a^11^. Y160A dramatically reduces its interaction with VPS39 and ameliorates its pro-apoptotic activity^15,19^. **c**–**e** SARS-CoV-2 ORF3a expression impaired ER ion homeostasis, as indicated by increased [Cl^–^]_ER_ (**c**) and [K^+^]_ER_ (**d**) and decreased [Ca^2+^]_ER_ (**e**). SARS-CoV-2 ORF3a and its mutants were transiently expressed in 293FT cells stably expressing ER-localized ratiometric sensors. The ion concentrations are indicated as the ratio of the mean intensities of the two fluorescent proteins^35^. **f** Schematic of the PiggyBac transposon and Tet-On system for inducible APEX2-tagged SARS-CoV-2 ORF3a expression. The HP138-puro plasmid contains PiggyBac terminal repeats flanking an EF1α promoter-driven cassette encoding rtTA and puromycin resistance (linked via a T2A self-cleaving peptide). Doxycycline (Dox) induces rtTA-dependent expression of APEX2-fused and GFP-tagged ORF3a (ORF3a-GFP-APEX2). GFP-expressing cells were used as a control. **g** TEM of 293FT cells expressing SARS-CoV-2 ORF3a-GFP-APEX2. APEX2-catalyzed DAB electron-dense precipitates (arrows) indicate ORF3a localization. Color annotations: green, CM (cell membrane); blue, Ly (lysosome) and AL (autolysosome); yellow, outer NE (nuclear envelope); red, ER (endoplasmic reticulum). Mito (mitochondria) are unlabeled. Scale bar, 200 nm. **h**–**j** Transient expression of SARS-CoV-2 ORF3a triggers the UPR. The three branches of the UPR were measured separately by co-transfection of reporter plasmids and SARS-CoV-2 ORF3a-encoding plasmids. RNA splicing of human *XBP1 by* IRE1 (**h**), translation of human ATF4 by PERK phosphorylation upon eIF2α (**i**), and cleaved ATF6 binding to its target DNA element as a transcription factor (**j**). The medium intensity of GFP and the percentage of GFP^+^ cells from miRFP670s^+^ cells were measured. In **c**–**e**, **h**–**j**, > 3000 miRFP670s^+^ cells were recorded per experiment, and *n* ≥ 3. The values are presented as the mean ± SD. n.s., no significant difference; *P < 0.05, **P < 0.01, ***P < 0.001, ****P < 0.0001 by one-way ANOVA or Student’s *t*-test.

We initially used the SARS-CoV-2 trans-complementation system^36^ to confirm the interaction between ORF3a and CLCC1. Endogenous CLCC1 was co-immunoprecipitated with ORF3a in Caco-2 cells infected with transcription- and replication-competent virus-like particles (trVLPs) of SARS-CoV-2 (Fig. 1a). Before performing a series of functional assays, we confirmed the interaction between SARS-CoV-2 ORF3a and CLCC1 in 293FT cells. Endogenous CLCC1 was immunoprecipitated with FLAG-tagged SARS-CoV-2 ORF3a but not with FLAG-tagged calnexin, an ER-localized membrane protein (Supplementary Fig. S1a). Subsequently, we expressed the FLAG-tagged SARS-CoV-2 ORF3a in 293FT cells expressing CLCC1-TurboID, enabling proximity labeling with biotin^37^. Following 15 min of treatment with exogenous biotin, the biotinylated SARS-CoV-2 ORF3a was captured from the cell lysate (Supplementary Fig. S1b). We subsequently performed a pull-down assay in vitro to determine the direct interaction between SARS-CoV-2 ORF3a and CLCC1 (Supplementary Fig. S1c).

Loss-of-function *CLCC1* disrupts ER ion homeostasis, which is characterized by increased [Cl^–^]_ER_ and [K^+^]_ER_ and decreased [Ca^2+^]_ER_^35^. To assess the effect of SARS-CoV-2 ORF3a on ER ion homeostasis, we measured ER luminal [Cl^–^], [K^+^], and [Ca^2+^] by flow cytometry in 293FT cells transiently expressing SARS-CoV-2 ORF3a and stably expressing genetically encoded ratiometric probes as previously reported^35^. Cells expressing SARS-CoV-2 ORF3a and its mutants were sorted by miRFP670s fluorescence protein using fluorescence-activated cell sorting (FACS) (Fig. 1b; Supplementary Fig. S1d). The transient expression of SARS-CoV-2 ORF3a led to increases in [Cl^–^]_ER_ (Fig. 1c) and [K^+^]_ER_ (Fig. 1d) but a decrease in [Ca^2+^]_ER_ (Fig. 1e), which is similar to previous observations in *CLCC1*-deficient cells^35^. We replaced the predicted signal peptide of SARS-CoV-2 ORF3a (amino acids 1–13) with that of calreticulin to promote its ER localization (ER-ORF3a, Fig. 1b; Supplementary Fig. S2). Similar to that of SARS-CoV-2 ORF3a, the expression of ER-ORF3a impaired [Cl^–^]_ER_, [K^+^]_ER_, and [Ca^2+^]_ER_ (Fig. 1c–e), suggesting that ER-localized SARS-CoV-2 ORF3a is sufficient to impair ER ion homeostasis. Like SARS-CoV-2 ORF3a and ER-ORF3a, two SARS-CoV-2 ORF3a mutations, Q116L^9^ and C133A^9,11^, significantly disrupted ER ion homeostasis (Fig. 1c–e; Supplementary Fig. S3a, b), indicating that the previously reported SARS-CoV-2 ORF3a ion selectivity and dimerization are not essential for SARS-CoV-2 ORF3a to impair ER ion homeostasis. Consistent with these findings, co-immunoprecipitation (co-IP) experiments confirmed the interactions of ER-ORF3a, SARS-CoV-2 ORF3a^Q116L^, and SARS-CoV-2 ORF3a^Y160A^ with endogenous CLCC1 (Supplementary Fig. S3c–e).

Given that the subcellular localization of ORF3a is still controversial, we performed transmission electron microscopy (TEM) and three-dimensional reconstruction to detect inducible APEX2-tagged SARS-CoV-2 ORF3a (Fig. 1f, g; Supplementary Video S1). Compared with those in the GFP control group (Fig. 1g (0)), 3,3′-diaminobenzidine (DAB) precipitates were observed on the cell membrane (Fig. 1g (1, 2)), lysosome (Fig. 1g (4)), autolysosome (Fig. 1g (1, 2, 3)), ER membrane (Fig. 1g (1, 5, 6)), and nuclear envelope (Fig. 1g (1, 3)). In addition, we detected a strong DAB signal in a cluster of the ER-like structure (Fig. 1g (7)). These results suggest that SARS-CoV-2 ORF3a has diverse functions in different organelles.

To confirm the activation of the UPR by SARS-CoV-2 ORF3a expression through direct measurement, we used three GFP-based reporter systems to monitor the UPR along the IRE1 branch (via mRNA splicing of *Xbp1*), the PERK branch (via ATF4 translation), and the ATF6 branch (via binding of ATF6 to target DNA sequences) with flow cytometry^38–40^. All three reporters responded to thapsigargin (Tg) or tunicamycin (Tm), which are ER stress inducers (Supplementary Fig. S4). The expression of SARS-CoV-2 ORF3a and ER-ORF3a activated the UPR in all three branches, as evidenced by increased median GFP intensity and GFP^+^ cell proportion (Fig. 1h–j), indicating that the expression of SARS-CoV-2 ORF3a leads to the ER UPR.

### SARS-CoV-2 ORF3a reshapes endomembranes

**Fig. 2:**
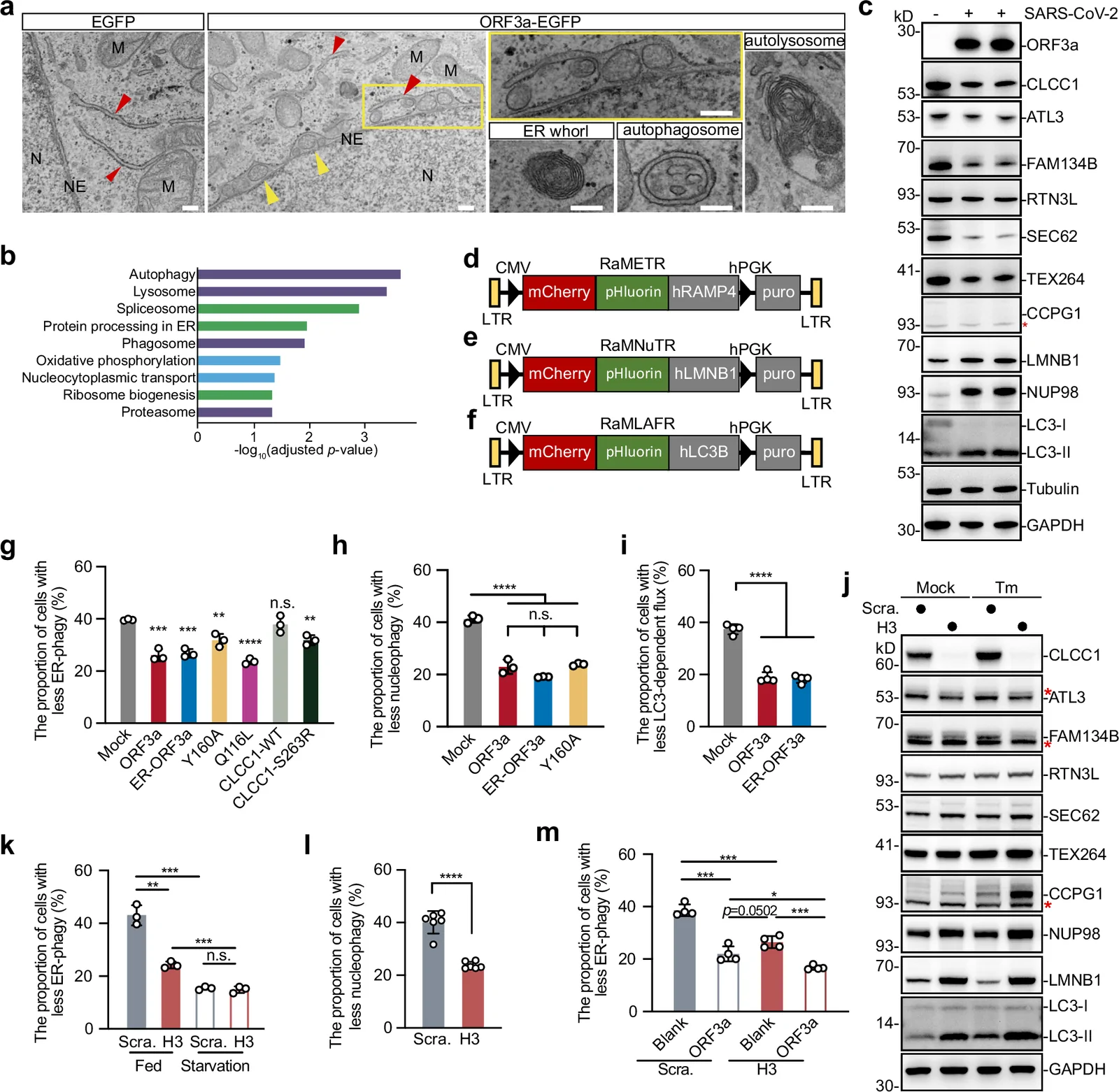
SARS-CoV-2 ORF3a expression, SARS-CoV-2 infection, and CLCC1 deficiency promote ER-phagy and nucleophagy. **a** TEM images of 293FT cells stably expressing SARS-CoV-2 ORF3a-EGFP or EGFP alone. Ribosome-bound ER structures are indicated with red arrows. ER budding, ER whorl, and autophagosomes were observed predominantly in SARS-CoV-2 ORF3a-EGFP-expressing 293FT cells. The yellow arrows and yellow boxes highlight the blebs amid the nuclear envelope. N nucleus, M mitochondria, NE nuclear envelope. **b** Gene enrichment analysis of 293FT cells stably expressing SARS-CoV-2 ORF3a-EGFP or EGFP alone. Upregulated KEGG categories are shown. **c** Western blot of the lysates from Vero cells infected with the SARS-CoV-2 virus of BetaCoV/Wuhan/IVDC-HB-01/2019 (C-Tan-HB01, GISAID accession no. EPI_ISL_402119) for 48 h. Vero cells without virus infection served as the negative control. Red asterisk, non-specific band. **d**–**f** Schematics of the ER-phagy reporter (RaMETR, **d**), nucleophagy reporter (RaMNuTR, **e**), and LC3-dependent autophagic flux reporter (RaMLAFR, **f**) based on the lentivirus strategy. The expression of the reporters is driven by the CMV promoter. The reporters are fusion proteins consisting of an internal control (mCherry), a pH-sensitive fluorescence protein (pHluorin), and an ER-resident ER-phagy substrate (human RAMP4, hRAMP4), a nucleophagy receptor (human Lamin B1, hLMNB1) or hLC3B. **g** SARS-CoV-2 ORF3a and CLCC1 mutants trigger ER-phagy. SARS-CoV-2 ORF3a, CLCC1, and their mutants were transiently expressed in 293FT cells stably expressing RaMETR. **h**, **i** SARS-CoV-2 ORF3a and its mutants trigger nucleophagy (**h**) and LC3-dependent autophagic flux (**i**). SARS-CoV-2 ORF3a and its mutants were transiently expressed in 293FT cells expressing RaMNuTR and RaMLAFR, respectively. **j** Western blot analysis of lysates from 293FT cells infected with *CLCC1* shRNA and treated with Tm (1 μg/mL for 18 h) or left untreated. H3, *CLCC1* shRNA. Scra., scrambled shRNA control. Red asterisks, non-specific bands. **k**, **l** CLCC1 deficiency promotes ER-phagy and nucleophagy. RaMETR and RaMNuTR were transiently expressed in *CLCC1*-knockdown cells (H3) and scrambled control cells (Scra.). After 6 h of RaMETR plasmid transfection, the cells were treated with EBSS solution (starvation) overnight to induce ER-phagy (**k**). **m** ER-phagy quantification in *CLCC1*-knockdown cells expressing SARS-CoV-2 ORF3a. RaMETR and ORF3a were transiently expressed in *CLCC1*-knockdown (H3) and scrambled control (Scra.) cells. Cells transfected with the empty vector (lacking ORF3a) served as a blank control. Scale bar in **a**, 200 nm. In **a**, > 30 cells per group were analyzed, *n* = 3. In **g**–**i** and **k**–**m**, > 3000 miRFP670s^+^ cells were recorded per experiment, with *n* ≥ 3. The values are presented as the mean ± SD. n.s., no significant difference; **P < 0.01, ***P < 0.001, ****P < 0.0001, by Student’s *t*-test and one-way ANOVA.

Considering the contrast limitation of DAB staining for determining membrane structure, we used TEM to examine the changes in endomembrane morphology of 293FT cells stably expressing EGFP (control) or SARS-CoV-2 ORF3a-EGFP. Cells expressing SARS-CoV-2 ORF3a-EGFP exhibited autolysosome and abnormal ER morphologies, including ribosome-bound autophagosomes as reported previously^16^ and concentric ER whorls, which were rarely observed in control cells (Fig. 2a). In addition, we documented the budding of the outer nuclear envelope into the cytoplasm and blebs between the outer and inner nuclear envelopes in cells expressing SARS-CoV-2 ORF3a-EGFP (Fig. 2a), suggesting that nucleophagy is induced by the expression of ORF3a.

We next performed RNA sequencing (RNA-seq) on stable cell lines expressing SARS-CoV-2 ORF3a-EGFP or an EGFP control. The differentially expressed genes (DEGs) were identified using DESeq2 (Supplementary Table S1), and enrichment analysis of the upregulated and downregulated DEGs was performed for the KEGG pathways (Supplementary Table S2). The upregulated genes were functionally enriched in catabolic pathways, including autophagy, lysosome, phagosome, and proteasome in the presence of SARS-CoV-2 ORF3a (Fig. 2b). For example, the expression of SARS-CoV-2 ORF3a led to the upregulation of autophagy receptors, including *SQSTM1* (encoding p62) and *TAX1BP1*, along with *ATG101*, *RAB7A*, *MAP1LC3B*, and *GABARAP* (Supplementary Table S1). Additionally, coupled with the downregulation of genes associated with the PI3K-Akt signaling pathway, the upregulated genes involved in the mTOR signaling pathway suggest a connection between SARS-CoV-2 ORF3a expression and autophagy (Supplementary Table S2). Furthermore, the upregulation of genes encoding lysosomal markers, such as *LAMP1*, and enzymes in lysosomes, such as *CTSA*, *CTNS*, *CTSD*, and *M6PR* (Supplementary Table S1), supported the adaptation of lysosomal functions to the expression of SARS-CoV-2 ORF3a. Interestingly, the upregulation of genes involved in oxidative phosphorylation and nucleocytoplasmic transport (Fig. 2b) suggests that SARS-CoV-2 ORF3a may also impair mitochondrial and nuclear envelope functions. Moreover, the upregulated genes were also functionally enriched in anabolic pathways, including spliceosome, protein processing in the ER, and ribosome biogenesis, indicating the potential disruption of mRNA splicing and protein synthesis and production in host cells by the expression of SARS-CoV-2 ORF3a, especially in the ER, an important component of the endomembrane system (Fig. 2b).

SARS-CoV-2 infection has been reported to burden the intracellular membrane^41–43^, while autophagy is a process through which host cells manage the damaged ER membrane and nuclear envelope^44^. We infected Vero cells with SARS-CoV-2 for 48 h and investigated whether SARS-CoV-2 infection promotes ER-phagy and nucleophagy (Fig. 2c). Virus-infected cells showed expression of ORF3a and reduced expression of CLCC1 and ER-phagy receptors, including ATL3, FAM134B, SEC62, and TEX264 (Fig. 2c)^45,46^. However, the ER-phagy receptor RNT3L did not change, and CCPG1 was not detected in Vero cells. Additionally, the expression of the nucleophagy receptor Lamin B1 (LMNB1)^47^ and nucleoporin NUP98 increased. Consistent with previous reports, SARS-CoV-2 infection enhanced LC3 lipidation (LC3 conversion from LC3-I to LC3-II)^15,16^ (Fig. 2c). Antibodies against ER-phagy and nucleophagy receptors were validated using shRNA-based gene knockdown or CRISPR/Cas9-based gene knockout cell lines (Supplementary Fig. S5). Thus, SARS-CoV-2 infection and ORF3a expression substantially burden ER membranes and nuclear envelopes.

### SARS-CoV-2 ORF3a causes ER-phagy, nucleophagy, and general LC3-dependent autophagic flux

To test whether ORF3a triggers ER-phagy, we optimized a previous ER-phagy reporter (eGFP-based ER Autophagy Tandem Reporter (EATR))^48,49^ by replacing eGFP with pHluorin, a pH-sensitive sensor that enhances the detection sensitivity in a cellular acidic environment, and by including mCherry as a ratiometric internal control (Fig. 2d). The ER-resident protein RAMP4, a substrate for ER-phagy, was fused with pHluorin and mCherry. We packed the resulting RatioMetric ER-phagy Tandem Reporter (RaMETR) into a lentivirus and generated a stable RaMETR reference line. Under naïve conditions, ~40% of cells with bright pHluorin fluorescence (indicating less ER-phagy) were classified as normal. During ER-phagy induced by Earle’s balanced salt solution (EBSS), this percentage decreased to < 10% within the same fluorescence gate (Supplementary Fig. S6a–c). Similarly, RaMETR responded to Torin 1 and Tm but not Brefeldin A (BFA) or Tg (Supplementary Fig. S6a–c).

To quantitatively analyze nucleophagy, we developed a RatioMetric Nucleophagy Tandem Reporter (RaMNuTR), which consists of mCherry, pHluorin, and LMNB1, a receptor for nucleophagy^47^ (Fig. 2e). Stably expressed RaMNuTR responded to Tm, BFA, EBSS, and Torin 1 but not to Tg (Supplementary Fig. S6d, e). In addition, bafilomycin A1 (BafA1), which neutralizes the lysosome^50^, strongly inhibited nucleophagy (Supplementary Fig. S6d, e). Live imaging of RaMNuTR further confirmed that nucleophagy was triggered by Torin 1 (Supplementary Fig. S6f).

To quantitatively monitor LC3 movement into acidic organelles, we generated a RatioMetric LC3-dependent Autophagic Flux Reporter (RaMLAFR). RaMLAFR comprises mCherry, pHluorin, and human LC3B (hLC3B) (Fig. 2f). RaMLAFR responded to EBSS, Torin 1, and Tg (Supplementary Fig. S6g, h).

Using these reporters, we examined the effects of SARS-CoV-2 ORF3a expression on ER-phagy, nucleophagy, and LC3-dependent autophagic flux in 293FT cells. The expression of SARS-CoV-2 ORF3a, ER-ORF3a, SARS-CoV-2 ORF3a^Q116L^, and SARS-CoV-2 ORF3a^Y160A^ triggered ER-phagy (Fig. 2g). The expression of a previously reported amyotrophic lateral sclerosis-associated loss-of-function mutant, S263R^35^, but not wild-type CLCC1 also led to ER-phagy, suggesting that the expression of SARS-CoV-2 ORF3a may cause loss of function of CLCC1 (Fig. 2g). Like SARS-CoV-2 ORF3a, ER-ORF3a and SARS-CoV-2 ORF3a^Y160A^ induced nucleophagy and LC3-dependent autophagic flux, as determined by our reporters (Fig. 2h, i). Thus, we conclude that the expression of SARS-CoV-2 ORF3a leads to ER-phagy and nucleophagy, independent of the key residues Q116 and Y160 in the ORF3a protein.

### CLCC1 deficiency causes ER-phagy and nucleophagy

To further demonstrate that CLCC1 deficiency triggers ER-phagy and nucleophagy, we generated *CLCC1* knockdown (H3) and scrambled control cells and examined the expression of ER-phagy receptors^45,46^ (Fig. 2j). Deficiency of CLCC1 led to changes in the expression of most of these receptors (decreased expression of ATL3 and FAM134B and increased expression of SEC62 and CCPG1), except for RTN3L and TEX264. Increased expression of LMNB1 and NUP98 was also detected in *CLCC1*-deficient cells (Fig. 2j). In addition, enhanced LC3 lipidation was observed in the deficient cells (Fig. 2j).

Upon the induction of ER stress by Tm, we detected increased expression of CLCC1 and CCPG1 and decreased expression of LMNB1 but little change in expression of the remaining proteins that we examined in naïve 293FT cells (Fig. 2j). Notably, CCPG1 expression significantly increased in *CLCC1*-deficient cells upon ER stress (Fig. 2j), which is consistent with a previous report that CCPG1 senses Tm-triggered unfolded protein accumulation in the ER lumen and promotes ER-phagy^51^.

We then used RaMETR to examine ER-phagy in the scrambled shRNA control (Scra.) and *CLCC1*-knockdown cells (H3). Compared to 43.1% ± 3.8% cells with normal ER-phagy in the control group, only 24.3% ± 1.3% cells had normal ER-phagy in the *CLCC1*-deficient cells (Fig. 2k), a result that was similar to that in cells expressing the ORF3a protein (Fig. 2g).

Given that CLCC1 deficiency induces ER-phagy, we examined whether EBSS-mediated starvation could amplify the differential ER-phagy response between *CLCC1*-knockdown cells and control cells. Upon EBSS treatment, the percentage of cells with less ER-phagy decreased to 15.4% ± 0.6% in the controls and 14.9% ± 1.2% in *CLCC1*-deficient cells, with no significant difference between them (Fig. 2k), suggesting that starvation masks the specific contribution of CLCC1 deficiency to ER-phagy.

Next, we expressed RaMNuTR in these two groups. We observed that 40.1% ± 4.3% of cells in the control group exhibited normal nucleophagy, which decreased to 23.8% ± 1.4% in *CLCC1*-knockdown cells (Fig. 2l), which was a similar level to that in cells expressing the ORF3a protein (Fig. 2h).

To test whether ORF3a impairs ER function through CLCC1, we overexpressed ORF3a in *CLCC1*-deficient cells and monitored the ER-phagy response. The percentage of cells with less ER-phagy decreased to 16.8% ± 0.9% in *CLCC1*-deficient cells expressing ORF3a, which was slightly greater than that in both the ORF3a expression group (22.0% ± 2.9%) and the *CLCC1* knockdown group (26.5% ± 2.3%) (Fig. 2m). Therefore, we conclude that both CLCC1 deficiency and ORF3a expression cause ER-phagy and nucleophagy.

### ORF3a/CLCC1 ratio determines their subcellular localization

**Fig. 3:**
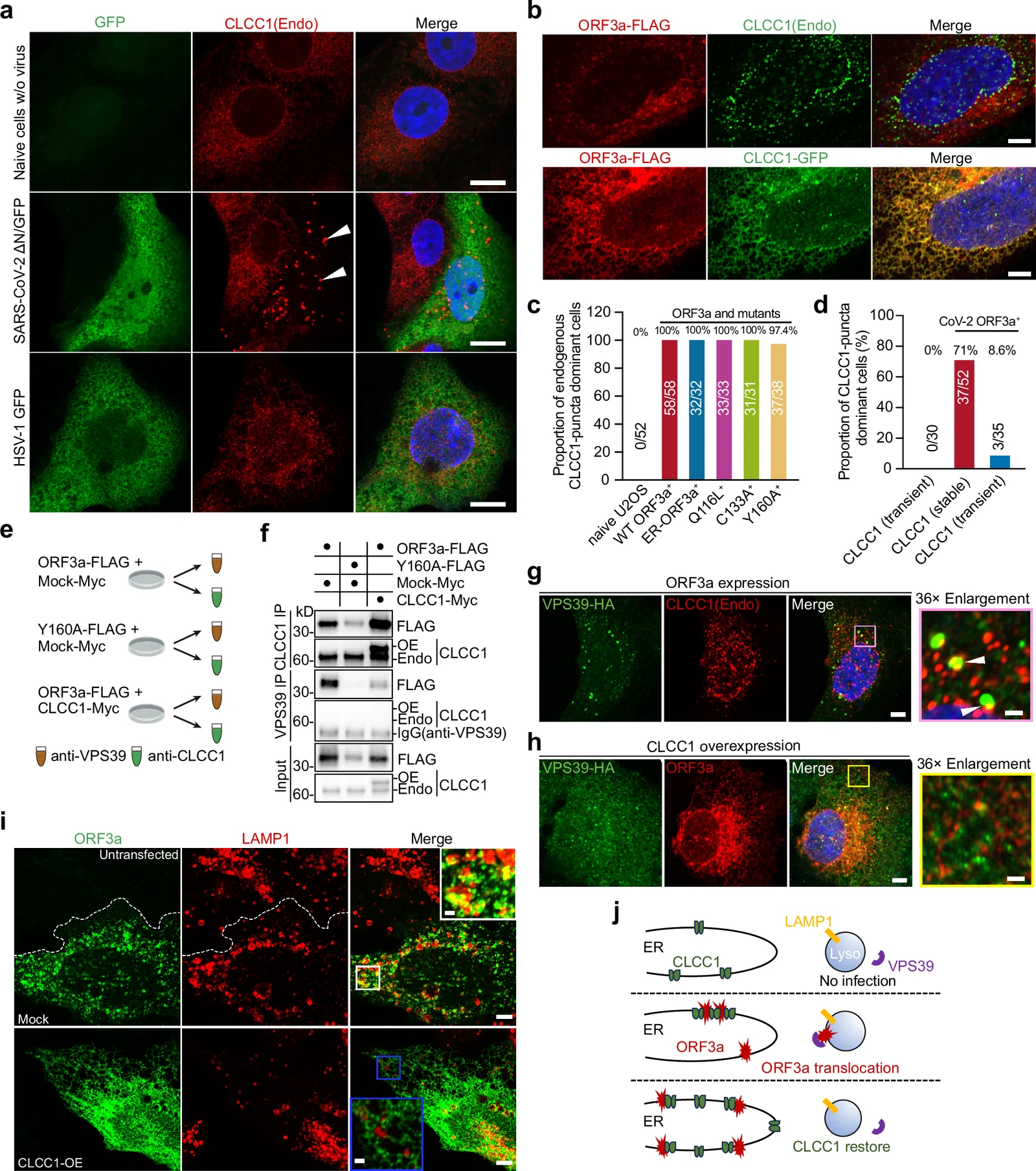
The ratio of CLCC1 to SARS-CoV-2 ORF3a controls their subcellular localization. **a** Immunofluorescence analysis of CLCC1 in Vero cells infected with SARS-CoV-2 ΔN/GFP trVLPs. Vero cells with HSV-1 GFP and without any virus infection served as negative controls. The arrows highlight the CLCC1 puncta in SARS-CoV-2 ΔN/GFP virus-infected cells. **b** Immunofluorescence analysis of CLCC1 and SARS-CoV-2 ORF3a in U2OS cells. FLAG-tagged SARS-CoV-2 ORF3a was transiently expressed in naïve U2OS cells (upper panels) and CLCC1-GFP-overexpressing U2OS cells (lower panels). SARS-CoV-2 ORF3a, endogenous CLCC1, and exogenous CLCC1 were stained with antibodies against FLAG, CLCC1, and GFP, respectively. **c**, **d** Quantitative analysis of CLCC1 puncta in U2OS cells in the presence of SARS-CoV-2 ORF3a and its mutants. The CLCC1 expression patterns were classified into puncta-dominant or tubule-dominant types. Naïve U2OS cells (**c**); U2OS cells with varying levels of CLCC1 expression (**d**). **e**, **f** Overexpression of CLCC1 inhibits SARS-CoV-2 ORF3a translocation to lysosomes. The co-IP assay design is shown in **f**. The lysates from 293FT cells co-expressing FLAG-tagged SARS-CoV-2 ORF3a and Myc-tagged human CLCC1 (hCLCC1) were divided equally and subjected to two co-IP experiments using the VPS39 antibody and CLCC1 antibodies. A plasmid lacking the h*CLCC1* gene was used as a mock control. The Y160A mutation significantly reduced the interaction of SARS-CoV-2 ORF3a with VPS39, which served as a negative control. **g** Immunofluorescence analysis of VPS39 with CLCC1 in U2OS cells stably expressing HA-tagged human VPS39. Endogenous CLCC1 and exogenous VPS39 were detected using anti-CLCC1 and anti-HA antibodies, respectively, following SARS-CoV-2 ORF3a transient expression. **h** Co-localization analysis of VPS39 with SARS-CoV-2 ORF3a. FLAG-tagged ORF3a and Myc-tagged CLCC1 were co-expressed in U2OS cells expressing HA-tagged human VPS39. ORF3a and VPS39 were stained with FLAG and HA antibodies, respectively. **i** Co-localization analysis of FLAG-tagged SARS-CoV-2 ORF3a and endogenous LAMP1. FLAG-tagged ORF3a was transiently expressed with or without CLCC1 in U2OS cells. Cells transfected with the empty vector served as mock controls. Insets show magnified regions indicated by colored boxes. The dashed line separates untransfected cells from ORF3a-expressing cells. **j** Illustration of how the subcellular localization patterns of CLCC1 and SARS-CoV-2 ORF3a are influenced by their expression ratios. In naïve cells, endogenous CLCC1 displays a dispersed pattern (upper). SARS-CoV-2 ORF3a recruits and saturates endogenous CLCC1, translocates to lysosomes and inhibits autolysosome formation by sequestering VPS39 (middle). CLCC1 overexpression results in the retention of SARS-CoV-2 ORF3a in the ER and the restoration of lysosomal function (lower). Scale bar in **a**, 10 μm; **b**, **g**–**i** 5 μm; in enlarged **g**–**i**, 1 μm. In **c** and **d** > 30 cells were counted per group of experiments.

Cells exposed to the SARS-CoV-2 virus undergo endomembrane remodeling^41,52^. We next explored whether the subcellular localization of CLCC1 changes during SARS-CoV-2-triggered ER remodeling. We validated the CLCC1 antibody by immunofluorescence staining using U2OS cells expressing shRNA (H3) targeting *CLCC1* (Supplementary Fig. S7a). Strikingly, endogenous CLCC1 formed puncta following SARS-CoV-2 ΔN/GFP trVLPs infection in Vero cells but not following Herpes simplex virus 1 infection (Fig. 3a). We also observed the same phenotype in Vero cells infected with the SARS-CoV-2 virus in a manner independent of the multiplicity of infection (MOI) (Supplementary Fig. S7b). In immunostaining experiments, we defined CLCC1 “dots” as endogenous, diffuse signals observed in naïve cells without viral infection (Fig. 3a; Supplementary Fig. S7c). In contrast, CLCC1 “puncta” referred to enlarged, high-intensity aggregates induced by viral infection (Fig. 3a; Supplementary Fig. S7b). Given that CLCC1 interacts with SARS-CoV-2 ORF3a, we next asked whether the expression of SARS-CoV-2 ORF3a is sufficient to induce CLCC1 puncta. Indeed, endogenous CLCC1 formed puncta in U2OS cells expressing SARS-CoV-2 ORF3a transiently (58/58), particularly around the nucleus (Fig. 3b). Conversely, the distribution of endogenous CLCC1 was dispersed in naïve U2OS cells without punctum formation (0/52) (Supplementary Fig. S7c). Moreover, endogenous CLCC1 puncta were also clearly observed in U2OS cells transiently expressing ER-ORF3a (32/32), SARS-CoV-2 ORF3a^Q116L^ (33/33), SARS-CoV-2 ORF3a^C133A^ (31/31), and SARS-CoV-2 ORF3a^Y160A^ (37/38) (Fig. 3c; Supplementary Fig. S7d). Despite a significant decrease in SARS-CoV-2 ORF3a^C133A^ expression, it induced CLCC1 punctum formation (Fig. 3c; Supplementary Fig. S7d). Thus, SARS-CoV-2 ORF3a expression induces clustering of endogenous CLCC1, which is independent of several previously reported key residues of ORF3a.

Notably, the CLCC1-positive puncta represented only a subset of SARS-CoV-2 ORF3a-positive puncta (Fig. 3b, upper panel), suggesting that SARS-CoV-2 ORF3a may saturate all available CLCC1 molecules, with a portion of ORF3a existing in a non-CLCC1-bound state. Furthermore, the CLCC1 expression level does not appear to be the determinant factor for CLCC1 punctum formation, as transient expression of exogenous CLCC1 alone did not induce punctum formation (Supplementary Fig. S7d), suggesting that increasing CLCC1 expression could restore its normal subcellular distribution. Indeed, the percentage of cells with CLCC1 puncta caused by SARS-CoV-2 ORF3a decreased to 71% (37/52) in the U2OS cells stably expressing CLCC1-GFP (Fig. 3d; Supplementary Fig. S7e). The remaining double-positive cells (15/52) exhibited co-localization of SARS-CoV-2 ORF3a and CLCC1 with a typical ER shape (Fig. 3b). Moreover, the transient expression of exogenous CLCC1, which led to higher expression than stably expressing CLCC1 in cells (Supplementary Fig. S7f, g), further decreased the proportion of cells with CLCC1 puncta to 8.6% (3/35) (Fig. 3d). Taken together, our results suggest that SARS-CoV-2 ORF3a hijacks CLCC1 to disrupt ER functions and that increasing levels of CLCC1 may attenuate SARS-CoV-2 ORF3a-mediated phenotypes.

SARS-CoV-2 ORF3a was reported to interact with VPS39, a subunit of the lysosomal tethering complex HOPS, to impair the function of lysosomes in a Y160-dependent manner^15^. We demonstrated that increasing CLCC1 expression resulted in the retention of SARS-CoV-2 ORF3a in the ER (Fig. 3b). Therefore, increasing the expression of CLCC1 may inhibit SARS-CoV-2 ORF3a translocation to the lysosome. Therefore, we transiently expressed CLCC1 with SARS-CoV-2 ORF3a and SARS-CoV-2 ORF3a^Y160A^ (Fig. 3e, f). Indeed, VPS39 co-immunoprecipitated with the wild-type protein but not the Y160A mutant. However, the interaction between VPS39 and SARS-CoV-2 ORF3a was less efficient when CLCC1 was overexpressed. Consistent with these findings, higher expression levels of CLCC1 resulted in increased co-immunoprecipitation of SARS-CoV-2 ORF3a with CLCC1. Moreover, CLCC1 did not co-immunoprecipitate with VPS39, ruling out the formation of the CLCC1–ORF3a–VPS39 ternary complex under these conditions.

However, the biochemical data above do not reflect the relative binding affinities between ORF3a–CLCC1 and ORF3a–VPS39. Immunofluorescence analysis revealed that SARS-CoV-2 ORF3a triggered the formation of both VPS39 aggregates and CLCC1 puncta. Importantly, VPS39 did not co-localize with CLCC1 (Fig. 3g; Supplementary Fig. S7h), supporting their distinct subcellular distributions. In addition, the lysosome marker LAMP1 did not co-localize with CLCC1 puncta in SARS-CoV-2 ORF3a-expressing cells (Supplementary Fig. S7i). When CLCC1 was overexpressed in U2OS cells expressing SARS-CoV-2 ORF3a, the formation of VPS39 aggregates was reduced (Fig. 3h). Moreover, SARS-CoV-2 ORF3a did not co-localize with LAMP1 when CLCC1 was overexpressed (Fig. 3i). Therefore, our results support the idea that increased CLCC1 expression results in the retention of SARS-CoV-2 ORF3a in the ER and the inhibition of SARS-CoV-2 ORF3a translocation to lysosomes (Fig. 3j).

### Evolutionarily conserved functions of beta-coronavirus ORF3a proteins in ER regulation

**Fig. 4:**
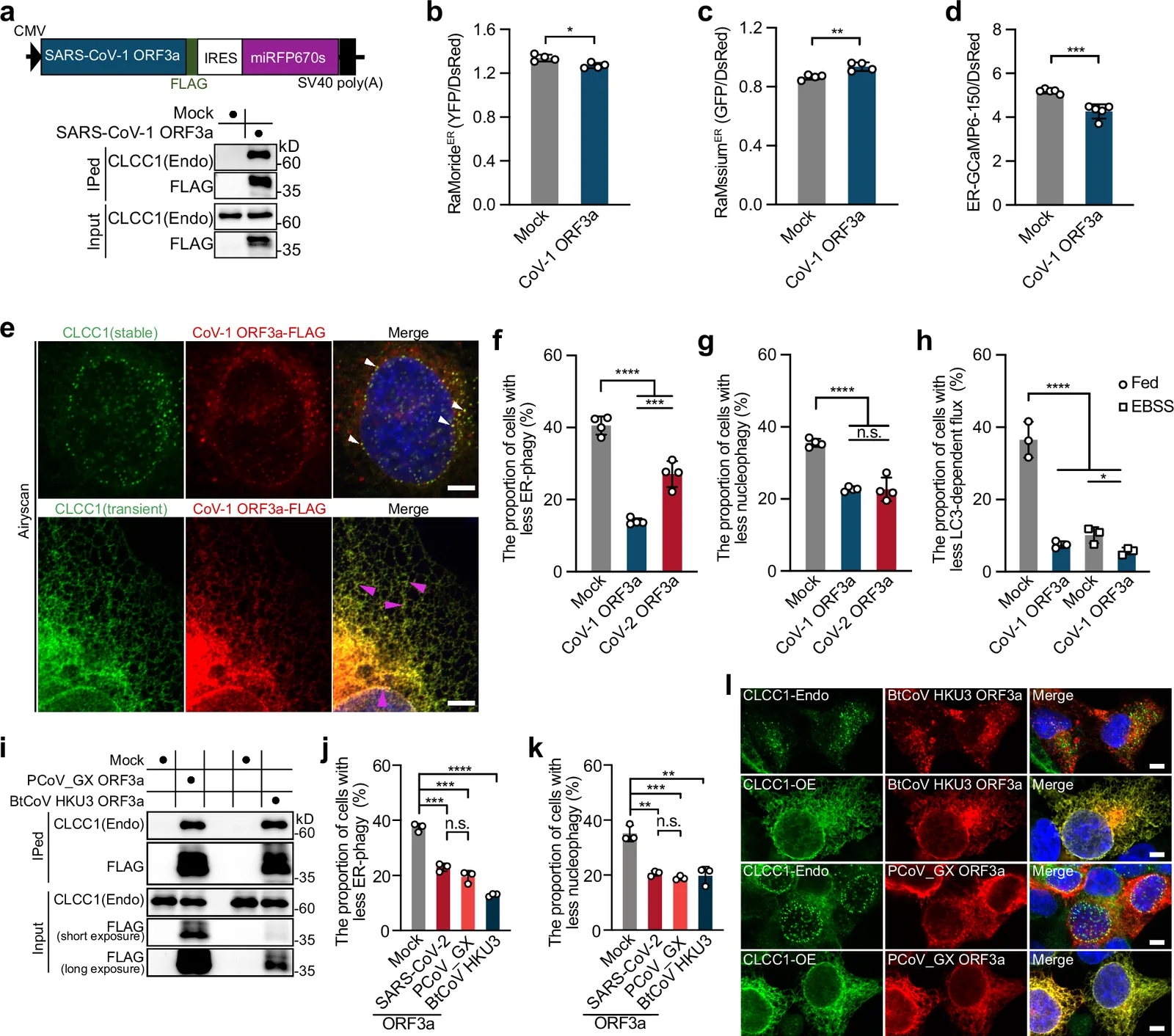
Conserved function of ORF3a among beta-coronaviruses in interactions with CLCC1. **a** SARS-CoV-1 ORF3a interacts with endogenous CLCC1. FLAG-tagged SARS-CoV-1 ORF3a was transiently expressed in 293FT cells and immunoprecipitated using M2 beads. Western blot analysis revealed CLCC1 and ORF3a-FLAG in the input and precipitate. **b**–**d** SARS-CoV-1 ORF3a impairs ER ion homeostasis. SARS-CoV-1 ORF3a was transiently expressed in 293FT cells, which stably expressed ER-localized ratiometric chloride (RaMoride^ER^, **b**), potassium (RaMssium^ER^, **c**) and calcium (ER-GCaMP6-150-DsRed, **d**) sensors. **e** Airyscan imaging of CLCC1 and SARS-CoV-1 ORF3a in U2OS cells. GFP-tagged CLCC1 was either stably (upper panel) or transiently (lower panel) expressed in U2OS cells and stained with a CLCC1 antibody. FLAG-tagged SARS-CoV-1 ORF3a was transiently expressed and immunostained with an anti-FLAG antibody. The white and purple arrows indicate the co-localization of CLCC1 and SARS-CoV-1 ORF3a in different patterns. **f**–**h** SARS-CoV-1 ORF3a triggers ER-phagy and nucleophagy and promotes LC3-dependent autophagic flux. SARS-CoV-1 ORF3a was transiently expressed in 293FT cells stably expressing RaMETR (**f**), RaMNuTR (**g**), and RaMLAFR (**h**). In **h**, the culture medium was replaced with EBSS 6 h post-transfection to induce autophagy overnight. In **i**, PCoV_GX ORF3a and BtCoV HKU3 ORF3a interact with endogenous CLCC1. The PCoV_GX ORF3a- and BtCoV HKU3 ORF3a-encoding genes were subcloned into the plasmid shown in **a**. FLAG-tagged PCoV_GX ORF3a and BtCoV HKU3 ORF3a were transiently expressed in 293FT cells and immunoprecipitated with M2 FLAG beads. CLCC1 and ORF3a were probed with CLCC1 antibody and FLAG antibody, respectively. **j**, **k** PCoV_GX ORF3a and BtCoV HKU3 ORF3a trigger ER-phagy and nucleophagy. PCoV_GX ORF3a and BtCoV HKU3 ORF3a were transiently expressed in 293FT cells stably expressing RaMETR (**j**) and RaMNuTR (**k**), which are the reporters of ER-phagy and nucleophagy, respectively. **l** Confocal imaging of CLCC1 and PCoV_GX ORF3a and BtCoV HKU3 ORF3a in 293FT cells. FLAG-tagged PCoV_GX ORF3a and BtCoV HKU3 ORF3a were transiently expressed and immunostained with an anti-FLAG antibody. Endogenous CLCC1 (CLCC1-endo) and overexpressed CLCC1 (CLCC1-OE) were stained with a CLCC1 antibody. Scale bar in **e** and **l**, 5 μm. For the FACS assay in **b**–**d**, **f**–**h**, and **j**, **k**, > 3000 miRFP670s^+^ cells were recorded per experiment, with *n* ≥ 3. The values are presented as the mean ± SD. n.s., no significant difference; *P < 0.05, **P < 0.01, ***P < 0.001, ****P < 0.0001, by Student’s *t-*test.

Because SARS-CoV-1 ORF3a shares 73% protein sequence similarity with SARS-CoV-2 ORF3a, we investigated whether they are functionally equivalent in hijacking CLCC1. SARS-CoV-1 ORF3a co-immunoprecipitated with endogenous CLCC1 (Fig. 4a). The expression of SARS-CoV-1 ORF3a increased [Cl^–^]_ER_ and [K^+^]_ER_ and decreased [Ca^2+^]_ER_ (Fig. 4b–d), similar to the results of the expression of SARS-CoV-2 ORF3a (Fig. 1). In addition, CLCC1 puncta were also observed in SARS-CoV-1 ORF3a-expressing cells, which was attenuated by increasing the overexpression of CLCC1 (Fig. 4e), as observed in SARS-CoV-2 ORF3a-expressing cells (Fig. 3b). The expression of SARS-CoV-1 ORF3a induced stronger ER-phagy than that of SARS-CoV-2 ORF3a (Fig. 4f), and triggered nucleophagy to a similar extent (Fig. 4g). In addition, SARS-CoV-1 ORF3a expression promoted LC3-dependent autophagic flux, which was not further worsened by EBSS starvation (Fig. 4h), suggesting that SARS-CoV-1 ORF3a expression saturated LC3. Importantly, EBSS treatment diminished the differential LC3-dependent autophagic flux, suggesting that LC3-dependent selective autophagy at the basal level, such as ER-phagy and nucleophagy, may not be accurately measurable under EBSS starvation conditions. Taken together, our results suggest that SARS-CoV-1 ORF3a also interacts with CLCC1 and affects CLCC1 functions to cause toxicity.

Many beta-coronaviruses have been identified, and as a conserved accessory protein^53,54^, ORF3a may affect CLCC1 functions in mammals in general. We synthesized two ORF3a-encoding genes from coronaviruses isolated from pangolin (PCoV_GX-P5L) and bat (BtCoV HKU3-1). Both PCoV_GX ORF3a (GenBank: QIA48633.1) and BtCoV HKU3 ORF3a (GenBank: AAY88867.1) interacted with CLCC1 (Fig. 4i) and induced ER-phagy (Fig. 4j) and nucleophagy (Fig. 4k). In addition, the expression of the PCoV_GX ORF3a and BtCoV HKU3 ORF3a induced CLCC1 puncta, which were reversed by transient CLCC1 expression (Fig. 4l). Thus, our results suggest that the common sequence among ORF3a from beta-coronaviruses may disrupt CLCC1 function and cause toxicity.

We next investigated interactions between the ORF3a homologs, VPS39 and CLCC1. SARS-CoV-1 ORF3a induced CLCC1 punctum formation but failed to interact with VPS39 or induce VPS39 aggregation, which is consistent with biochemical evidence indicating that these proteins do not interact (Supplementary Fig. S8a, b). In contrast, both the PCoV_GX ORF3a and BtCoV HKU3 ORF3a interacted with VPS39, inducing VPS39 aggregation, and this effect was attenuated by CLCC1 overexpression (Supplementary Fig. S8a, c, d). These findings demonstrate that beta-coronavirus ORF3a proteins play evolutionarily conserved roles in impairing ER homeostasis but exhibit functional divergence in lysosomal regulation.

### CLCC1 overexpression attenuates ORF3a-mediated pathological effects

**Fig. 5:**
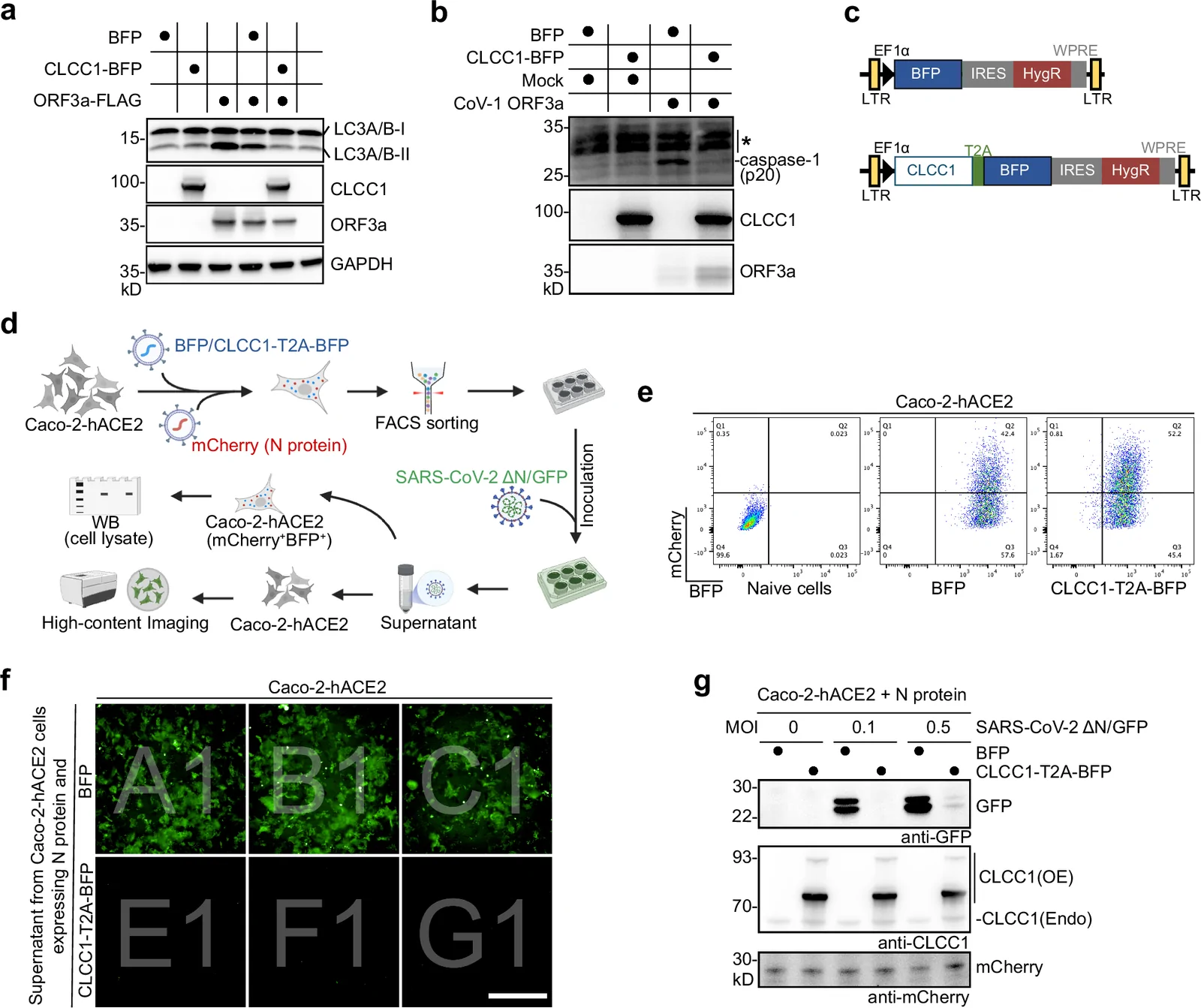
Increased CLCC1 expression rescues ORF3a toxicity and inhibits coronavirus propagation. **a** CLCC1 overexpression ameliorates LC3 lipidation triggered by SARS-CoV-2 ORF3a. BFP-tagged CLCC1 or BFP alone was co-transfected with FLAG-tagged SARS-CoV-2 ORF3a in 293FT cells. Individual plasmids were used alone as controls. CLCC1-BFP and ORF3a were probed with CLCC1 and FLAG antibodies, respectively. **b** Increased CLCC1 expression inhibits caspase-1 cleavage induced by SARS-CoV-1 ORF3a. BFP-tagged CLCC1 or BFP alone was co-transfected with FLAG-tagged SARS-CoV-1 ORF3a in 293FT cells. A plasmid lacking the SARS-CoV-1 ORF3a-encoding cassette was used as a mock control. The p20 component indicated the activation of caspase-1. CLCC1-BFP and ORF3a were probed with CLCC1 and FLAG antibodies, respectively. Asterisk, the non-specific bands. **c** Schematic of the lentiviral CLCC1 overexpression construct. The EF1α promoter drives the co-expression of CLCC1 and BFP via a T2A self-cleaving peptide with hygromycin resistance for stable cell selection. BFP-expressing cells served as negative controls. HygR hygromycin resistance. **d** Experimental workflow for CLCC1 functional analysis in coronavirus propagation. ACE2-stabilized Caco-2 cells underwent sequential lentiviral transduction for N protein and CLCC1 expression. FACS-sorted mCherry^+^ BFP^+^ cells were used to generate SARS-CoV-2 ΔN/GFP virions, and the supernatants were collected for downstream analysis. Created in BioRender. https://BioRender.com/pq6wq0l. **e** FACS sorting strategy for dual-positive Caco-2-hACE2 cells. mCherry fluorescence indicated N protein expression, with naïve cells used for gating calibration. **f** High-content imaging of SARS-CoV-2 ΔN/GFP infection in Caco-2-hACE2 cells. Supernatants from BFP- or CLCC1-T2A-BFP-expressing cells were added at equivalent volumes for 48 h. Scale bar, 500 μm. **g** Immunoblot analysis of SARS-CoV-2 ΔN/GFP-infected Caco-2-hACE2 (mCherry^+^ BFP^+^) lysates. Supernatants from BFP- or CLCC1-T2A-BFP-expressing cells were applied at the indicated MOIs.

A recent study reported that CLCC1 silencing triggers a homeostatic UPR to prevent cell death^55^. However, CLCC1 deficiency causes ER-phagy and nucleophagy (Fig. 2) and neuron loss in mutant mice^35^, suggesting that increased CLCC1 expression may counteract SARS-CoV-2 ORF3a toxicity. To prove this effect, we used a Tet-On system for inducible CLCC1 expression alongside transient ORF3a transfection. Inducible CLCC1-GFP (but not the GFP control) also significantly reduced SARS-CoV-2 ORF3a-induced LC3 lipidation (Supplementary Fig. S9). Consistent with these findings, transient co-expression of CLCC1 with SARS-CoV-2 ORF3a similarly suppressed LC3 lipidation (Fig. 5a). Together, these results support a protective role for CLCC1 against ORF3a-induced autophagy pathology.

Consistent with previously reported SARS-CoV-1 ORF3a-mediated NLRP3 inflammasome activation upstream of caspase-1 cleavage^17,18^, we observed enhanced caspase-1 cleavage (p20 fragment) upon SARS-CoV-1 ORF3a expression. This effect was suppressed by CLCC1 co-expression (Fig. 5b), highlighting the role of CLCC1 in attenuating viral-induced inflammatory responses.

### Viral restriction is mediated by CLCC1 sequestration of ORF3a

We hypothesized that CLCC1 overexpression may block coronavirus propagation by retaining ORF3a in the ER, given that the lysosomal localization of ORF3a promotes SARS-CoV-2 egress^13^. Using the SARS-CoV-2 trans-complementation system^36^, we assessed viral replication in CLCC1-overexpressing Caco-2 cells stably expressing hACE2. Cells underwent sequential lentiviral transduction for viral N gene delivery and CLCC1 overexpression (Fig. 5c–e). Following FACS, the cells were challenged with SARS-CoV-2 ΔN/GFP trVLPs. CLCC1 overexpression decreased infectious progeny production in secondary infection assays (Fig. 5f) and reduced intracellular SARS-CoV-2 ΔN/GFP trVLP replication (Fig. 5g).

### Identification of the interaction interface between SARS-CoV-2 ORF3a and CLCC1

**Fig. 6:**
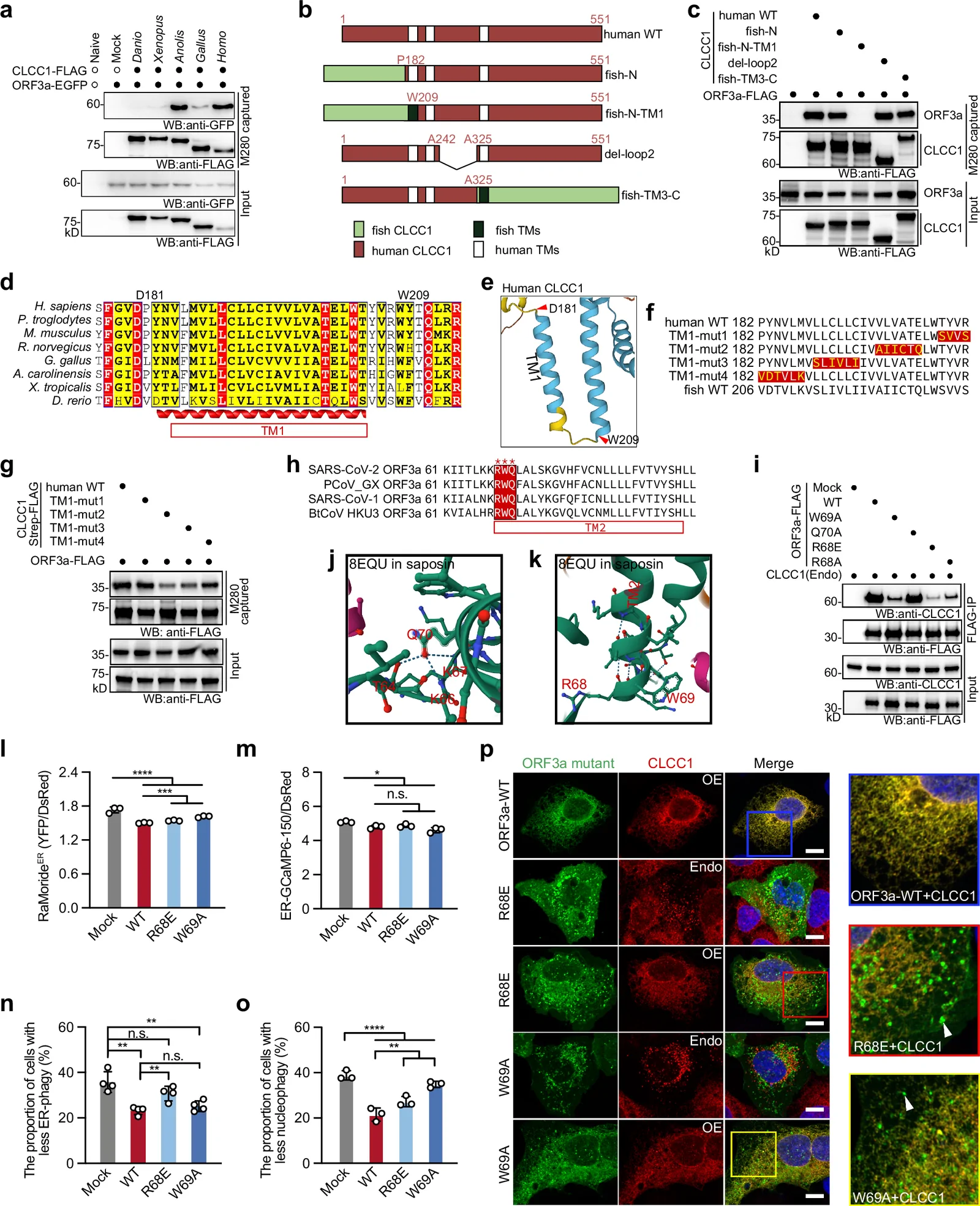
Interface of the SARS-CoV-2 ORF3a and CLCC1 interaction. **a** SARS-CoV-2 ORF3a interacts with CLCC1 in certain species. *Danio rerio* (XM_002667211.5), *Xenopus tropicalis* (XM_002932173.5), *Anolis carolinensis* (XM_003223596.3) and *Gallus gallus* (XM_422186.6) *CLCC1* were synthesized and codon optimized. All CLCC1 proteins, which were fused with a C-terminal Strep-FLAG-Strep tag, were co-expressed with GFP-tagged SARS-CoV-2 ORF3a, captured with M280 streptavidin beads, and subjected to western blot. CLCC1 and ORF3a proteins were probed with FLAG and GFP antibodies, respectively. **b**, **c** TM1 of human CLCC1 is required for SARS-CoV-2 ORF3a binding. The design of CLCC1 mutants with swapped domains is shown in **b**. CLCC1 mutants tagged with Strep-FLAG-Strep were co-expressed with SARS-CoV-2 ORF3a-FLAG, captured with M280 streptavidin beads, and subjected to western blot (**c**). The expression levels of CLCC1 and ORF3a were detected with a FLAG antibody. **d**, **e** Illustration of the CLCC1 TM1 sequence and structure. Protein sequence alignment of CLCC1 TM1 across species (**d**). The prediction of TM1 and TM2 of human CLCC1 by AlphaFold2 (**e**). **f**, **g** Residue substitutions in TM1 of human CLCC1 weaken its binding to SARS-CoV-2 ORF3a. Chimeric CLCC1 proteins tagged with Strep-FLAG-Strep were co-expressed with SARS-CoV-2 ORF3a-FLAG, captured with M280 streptavidin beads, and subjected to western blot. **h** Alignment of ORF3a protein sequences from four beta-coronaviruses. The part of ORF3a encoded by SARS-CoV-2, SARS-CoV-1, PCoV_GX-P5L, and BtCoV HKU3-1 is shown. TM2 boundary, red box; RWQ motif, red asterisk. **i** Residue substitutions in TM2 of SARS-CoV-2 ORF3a reduce its binding to endogenous CLCC1. FLAG-tagged SARS-CoV-2 ORF3a mutants were transiently expressed in 293FT cells, immunoprecipitated with anti-FLAG nanobody-conjugated beads, and subjected to western blot. ORF3a and CLCC1 were probed with anti-FLAG and anti-CLCC1 antibodies, respectively. **j**, **k** Cyro-EM structures of TM2 of SARS-CoV-2 ORF3a in a Saposin A nanodisc (PDB: 8EQU^10^). The residues R68, W69, and Q70 are highlighted in red. **l**–**o** Quantitative analysis of the effects of ORF3a mutants on ER ion homeostasis and autophagy. The wild-type and mutant SARS-CoV-2 ORF3a were transiently expressed in 293FT cells harboring ratiometric probes to measure [Cl^–^]_ER_ (**l**), [Ca^2+^]_ER_ (**m**), ER-phagy (**n**), and nucleophagy (**o**). > 3000 miRFP670s^+^ cells were recorded per experiment, with *n* ≥ 3. The values are presented as the mean ± SD. n.s., no significant difference; *P < 0.05, **P < 0.01, ***P < 0.001, ****P < 0.0001, by Student’s *t-*test. **p** Co-localization analysis of ORF3a and its mutants with CLCC1 in U2OS cells. FLAG-tagged ORF3a and its mutants were co-transfected with or without CLCC1. Co-localization between ORF3a-WT and overexpressed CLCC1 served as the positive control. ORF3a and CLCC1, including endogenous and overexpressed CLCC1, were detected using the appropriate antibodies. Endo endogenous; OE overexpressed. Scale bar, 10 μm.

CLCC1 proteins are evolutionarily conserved across species from the N-terminus to the third transmembrane domain (TM3)^35^. However, our co-IP experiments revealed that SARS-CoV-2 ORF3a strongly interacts with human CLCC1 and *Anolis* Clcc1 but weakly interacts with *Gallus* Clcc1 and does not interact with *Danio* (fish) or *Xenopus* Clcc1 (Fig. 6a). To identify the interaction interface between ORF3a and human CLCC1, we conducted swapping experiments with chimeric or truncated human CLCC1 (Fig. 6b). Truncated CLCC1 lacking the 2nd loop (del-loop2), chimeric CLCC1 that was generated by replacing human CLCC1 soluble N-terminus with the fish N-terminus (fish-N), and chimeric CLCC1 that was generated by replacing the human CLCC1 TM3 to the C-terminus with that of fish (fish-TM3-C) maintained the interaction with SARS-CoV-2 ORF3a (Fig. 6c). In contrast, chimeric CLCC1 that was generated by replacing the human CLCC1 N-terminus to the first transmembrane domain (TM1) with that of fish (fish-N-TM1) abolished the interaction (Fig. 6c), pinpointing that the P182–W209 fragment of CLCC1 (TM1) is crucial for the interaction, which was predicted as an alpha helix by AlphaFold2 (Fig. 6d, e). We generated chimeric CLCC1 proteins with four sequential residue substitutions spanning the P182–R208 region (Fig. 6f), in which substitutions replaced the corresponding residues in CLCC1 with those in fish. The substitutions CLCC1^SLIVLI^ (TM1-mut3) and CLCC1^AIICTQ^ (TM1-mut2) decreased their binding ability to SARS-CoV-2 ORF3a, whereas CLCC1^SVVS^ (TM1-mut1) and CLCC1^VDTVLK^ (TM1-mut4) strongly increased binding (Fig. 6g), further confirming that CLCC1 TM1 is crucial for SARS-CoV-2 ORF3a interaction.

Considering the similar topological structures of CLCC1^35^ and SARS-CoV-2 ORF3a^9,10^, we hypothesized that the interaction interface of SARS-CoV-2 ORF3a is located mainly in its N-terminal domain and TMs (Supplementary Fig. S10a). Compared with SARS-CoV-2 ORF3a and its N-terminal deletion (del-N), deletions of TM1 and TM2 (del-TM1–2) and of TM2 and TM3 (del-TM2–3) impaired the interaction (Supplementary Fig. S10b). On the basis of the previously reported cryo-electron microscopy (cryo-EM) structure of SARS-CoV-2 ORF3a^9,10^ and our data, we hypothesized that the N-terminus of TM2 is critical for this interaction. We focused on the RWQ motif at the beginning of TM2, which is conserved among SARS-CoV-2 ORF3a, SARS-CoV-1 ORF3a, PCoV_GX ORF3a, and BtCoV HKU3 ORF3a (Fig. 6h) and has been demonstrated to interact with CLCC1 (Fig. 4). To gain further insight into the critical residues in the RWQ motif crucial for the interaction, we generated five mutants of SARS-CoV-2 ORF3a (RWQ/AAA, W69A, R68E, R68A, and Q70A). Compared with Q70A, the other four mutations impaired the interaction between SARS-CoV-2 ORF3a and endogenous CLCC1 (Fig. 6i; Supplementary Fig. S10b). By reanalyzing the previously reported cryo-EM structure of ORF3a^10^, we determined that Q70 is embedded within the ORF3a homodimer, forming hydrogen bonds with T64, K66, and K67 (Fig. 6j). Moreover, R68 and W69 are positioned on the outer surface of the ORF3a homodimer (Fig. 6k), providing accessibility for interaction with CLCC1 TM1. Therefore, we conclude that the TM1 of CLCC1 and the TM2 of SARS-CoV-2 ORF3a form a dominant interface, facilitating their interaction.

We next investigated whether these mutations, including R68E and W69A, change the functions of ORF3a. R68E and/or W69A slightly ameliorate the [Cl^–^]_ER_, ER-phagy, and nucleophagy but not [Ca^2+^]_ER_ (Fig. 6l–o). We hypothesized that the transient expression of the mutants still blocks endogenous CLCC1 because their high dosage is sufficient to saturate all CLCC1 proteins. Indeed, ORF3a^R68E^ and ORF3a^W69A^ promoted the punctum formation of endogenous CLCC1 in U2OS cells (Fig. 6p). Interestingly, when CLCC1 was overexpressed in ORF3a^R68E^- and ORF3a^W69A^-expressing U2OS cells, the ORF3a signal was partly co-localized with CLCC1, and some proportion of the mutant ORF3a exhibited an aggregate form, indicating that R68E and W69A confer the ability of ORF3a to escape from CLCC1 retention in the ER (Fig. 6p).

### SARS-CoV-2 ORF3a causes pathologies in the mouse brain

Investigation of the pathologies mediated by the ORF3a‒CLCC1 interaction in vivo is challenging as suitable readouts are lacking in most cell types. Given that CLCC1 deficiency leads to specific neuropathologies, if ORF3a causes neuropathology by hijacking CLCC1 in the brain, the pathological features in ORF3a-expressing brains should recapitulate those observed in CLCC1-mutant mice. Moreover, whether ORF3a expression in the mouse brain can mimic relevant clinical symptoms remains to be determined.

We first examined the punctum formation of endogenous CLCC1 induced by ORF3a in SH-SY5Y cells. These results were consistent with observations in 293FT, Caco-2, Vero, and U2OS cells (Supplementary Fig. S11a), suggesting that ORF3a may similarly contribute to phenotypes in the brain.

**Fig. 7:**
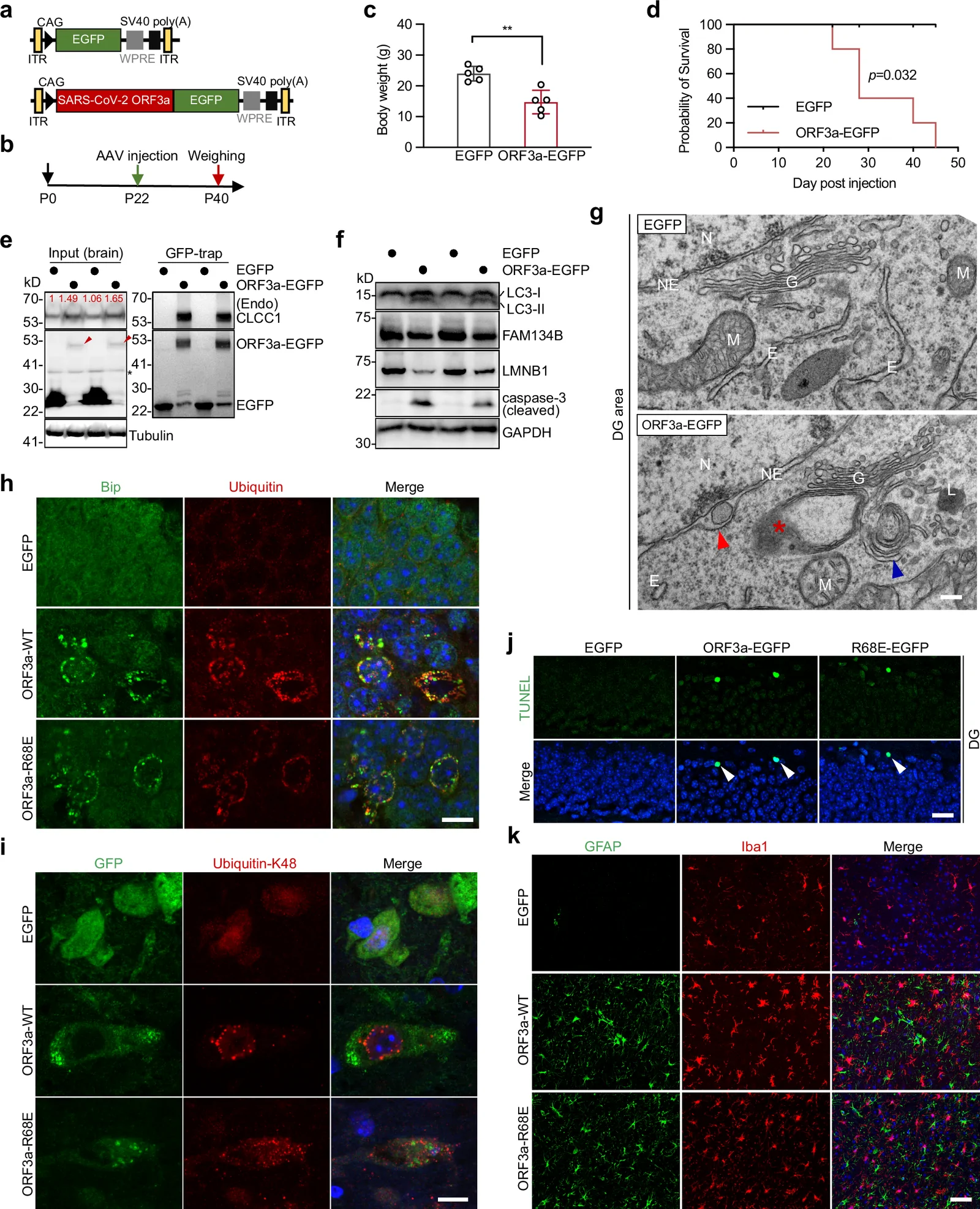
SARS-CoV-2 ORF3a causes pathogenesis in mice. **a** AAV-PHP.eB-based transgene delivery strategy involving retro-orbital injection in mice. EGFP-tagged SARS-CoV-2 ORF3a and EGFP were driven by a CAG promoter. **b**–**d** SARS-CoV-2 ORF3a expression leads to weight loss and mortality in mice. The wild-type mice were injected with AAV-PHP.eB at postnatal day 22 (P22) and weighed at P40 (**b**). The mice were sacrificed when substantial weight loss (**c**) and movement impairment (**d**) were observed. The dose of AAV-PHP. eB, 1 × 10^11^ vg/20 g mice. **e,** **f** SARS-CoV-2 ORF3a interacts with endogenous CLCC1 and induces LC3 lipidation, ER-phagy, nucleophagy, and apoptosis in mouse brains. Four mice were sacrificed 28 days post AAV-PHP.eB injection. Brain lysates were prepared for co-IP experiments (**e**) and immunoblot analysis (**f**). In **e**, GFP-trap beads captured the EGFP-tagged SARS-CoV-2 ORF3a and EGFP. Red arrows indicate EGFP-tagged SARS-CoV-2 ORF3a as input. The relative expression level of endogenous CLCC1 was calculated and normalized (**e**). In **f**, antibodies against LC3, FAM134B, LMNB1, and caspase-3 were used to detect LC3-I/LC3-II conversion, ER-phagy, nucleophagy, and caspase-3-dependent apoptosis, respectively. **g** TEM images of hippocampal regions expressing SARS-CoV-2 ORF3a-EGFP or EGFP. Red arrow, the bleb amid NE; red asterisk, the autolysosome; blue arrow, ER whorl-like membrane stacks. N nucleus, M mitochondria, NE nuclear envelope, G Golgi apparatus; E endoplasmic reticulum, L lysosome. **h**, **i** Immunofluorescence analysis of mouse brains expressing SARS-CoV-2 ORF3a-EGFP and EGFP. The mice were sacrificed 20 days post AAV-PHP.eB administration. ER stress, misfolded protein accumulation, and protein degradation were assessed using staining for Bip, ubiquitin, and K48-linked ubiquitin, respectively, in the dentate gyrus and olfactory bulbs. **j** TUNEL assay of the hippocampal dentate gyrus in mice expressing SARS-CoV-2 ORF3a-EGFP vs EGFP control. **k** Neuroinflammatory profiling in thalamic regions of ORF3a-EGFP-expressing mice. Microglial activation (Iba1) and astrogliosis (GFAP) were assessed by immunostaining. Scale bar, 200 nm in **g**; 5 μm in **h** and **i**; 20 μm in **j**; 50 μm in **k**. In **b**–**d**, *n* = 5. The values are presented as the mean ± SD. **P < 0.01, by Student’s *t*-test (**c**) and the Mantel‒Cox test (**d**).

To assess the in vivo relevance of the ORF3a-CLCC1 axis, we expressed ORF3a in the mouse brain using AAV delivery and evaluated neurological and cellular outcomes. Here, we used AAV-PHP.eB, an AAV serotype with high transduction efficiency in the central nervous system^56^, and inserted *EGFP* and the *SARS-CoV-2 ORF3a-EGFP* expression cassette driven by the CAG promoter (Fig. 7a). The resulting AAV-PHP.eB viruses were injected into wild-type mice via retro-orbital injection (1 × 10^11^ vg/mouse) at postnatal day 22 (P22) (Fig. 7b). Both EGFP and SARS-CoV-2 ORF3a-EGFP were robustly expressed in the brain 20 days post injection (Supplementary Fig. S11b). Compared with the mice expressing EGFP, the mice expressing SARS-CoV-2 ORF3a-EGFP exhibited reduced body weight and movement impairment (Fig. 7c; Supplementary Video S2). All the mice expressing SARS-CoV-2 ORF3a-EGFP died within 2 months after AAV injection (Fig. 7d).

The interaction between SARS-CoV-2 ORF3a and CLCC1 in the brain was confirmed by immunoprecipitation with GFP nanobody-conjugated beads in mice injected with AAV-PHP.eB expressing SARS-CoV-2 ORF3a-EGFP but not EGFP (Fig. 7e). Enhanced LC3 lipidation, degradation of FAM134B and LMNB1, and cleavage of caspase-3 were detected in mouse brains expressing SARS-CoV-2 ORF3a-EGFP but not EGFP, indicating that mouse brains expressing SARS-CoV-2 ORF3a-EGFP undergo ER-phagy, nucleophagy, and apoptosis (Fig. 7f). We observed that CLCC1 was upregulated in mouse brains expressing SARS-CoV-2 ORF3a-EGFP but not EGFP (Fig. 7e), possibly due to a compensatory mechanism.

SARS-CoV-2 ORF3a expression altered ER morphology and nuclear envelope structure in vitro (Figs. 1g and 2a). To validate these changes in vivo, we applied TEM to mouse brains expressing SARS-CoV-2 ORF3a-EGFP or EGFP. We observed blebs amid the nuclear envelope, autolysosome, and ER whorl-like membrane stacks in the mouse hippocampus expressing SARS-CoV-2 ORF3a-EGFP but not in the EGFP controls (Fig. 7g).

*CLCC1* loss of function has been demonstrated to induce ER stress and unfolded protein accumulation in diseased neurons^35,57^. If SARS-CoV-2 ORF3a hijacks CLCC1 to impair CLCC1 function, we would expect to observe these phenotypes in the SARS-CoV-2 ORF3a-expressing brain. Bip upregulation, ubiquitinated protein accumulation, and enhanced K48-ubiquitin signaling were indeed detected in the brains of mice expressing SARS-CoV-2 ORF3a-EGFP but not EGFP, including the olfactory bulb, cortex, hippocampus, thalamus, and cerebellum (Fig. 7h, i; Supplementary Fig. S11c). TUNEL staining also revealed neuronal death in the brains of mice expressing SARS-CoV-2 ORF3a-EGFP (Fig. 7j). Because neuronal loss may change the microenvironment that is sensed by surrounding glial cells, we investigated changes in immune status in the brain. The number of GAFP^+^ astrocytes and Iba1^+^ microglia increased significantly in the thalamic region of the brains of mice expressing SARS-CoV-2 ORF3a-EGFP, indicating that strong neuroimmune responses occurred (Fig. 7k).

We next investigated the biological effects of ORF3a^R68E^ because R68E reduced the binding ability of ORF3a to CLCC1 (Fig. 6). Even though ORF3a^R68E^ exhibited weak binding with endogenous CLCC1, the body weight did not increase to the same level as that of the EGFP group (Supplementary Fig. S11d, e). In addition, ORF3a^R68E^ led to ER stress, cell death, and robust immune responses in the brain (Fig. 7h–k). As shown in Fig. 6p, the constitutive expression of mutant ORF3a driven by the CAG promoter may have saturated CLCC1.

These findings underscore the significant pathological implications of ORF3a expression in vivo, potentially reflecting severe neurological outcomes associated with SARS-CoV-2 infection in patients.

## Discussion

CLCC1 has been reported in multiple studies to be among the top hits of SARS-CoV-2 ORF3a-binding host proteins^31–34^. We hypothesize that the chloride homeostasis and ER functions controlled by CLCC1 may be critical for SARS-CoV-2 pathogenicity and COVID-19 pathology. Recently, the SARS-CoV-2 E protein has been shown to elevate intracellular chloride concentrations, impair the airway epithelial barrier, and exacerbate airway inflammation^58^, further highlighting the role of chloride homeostasis in disease pathologies. Here, we confirmed CLCC1–ORF3a binding in vitro and in vivo (Figs. 1a, 7e; Supplementary Fig. S1a–c) and then demonstrated that SARS-CoV-2 ORF3a impairs ER ion homeostasis, as evidenced by increased [Cl^–^]_ER_ and [K^+^]_ER_ and decreased [Ca^2+^]_ER_ (Fig. 1c–e) and activation of UPRs in vitro (Fig. 1h–j) and ER stress in vivo (Fig. 7h, i; Supplementary Fig. S11c), suggesting that SARS-CoV-2 ORF3a may hijack CLCC1 to cause ER dysfunction.

Whether SARS-CoV-2 ORF3a acts as a viroporin to mediate ionic currents to induce phenotypes remains controversial^9,10^. Even if SARS-CoV-2 ORF3a functions as an ion channel, SARS-CoV-2 ORF3a^Q116L^ alters ion selectivity and disrupts ER homeostasis similar to the original SARS-CoV-2 ORF3a, suggesting that SARS-CoV-2 ORF3a may modulate ER luminal ion concentration and ER function through interactions with host proteins, such as CLCC1, rather than through its channel activity (Fig. 1c; Supplementary Fig. S3a, b).

In addition, both SARS-CoV-2 ORF3a expression and CLCC1 deficiency led to ER-phagy and nucleophagy (Fig. 2), and overexpression of CLCC1 ameliorated LC3 lipidation (Fig. 5a), positioning CLCC1 downstream of ORF3a but upstream of LC3 in this cascade. First, the reduced CLCC1 expression level in virus-infected cells (Figs. 1a and 2c) is unlikely to be caused by ORF3a alone but by other viral proteins or the combination of viral factors. In contrast, CLCC1 expression levels remained stable in 293FT cells expressing SARS-CoV-2 ORF3a alone (Supplementary Figs. S1a and 9b) and were even higher in mouse brains in the presence of SARS-CoV-2 ORF3a (Fig. 7e), indicating that the ER dysfunction attributed to SARS-CoV-2 ORF3a is not due to reduced CLCC1 expression levels. Notably, the channel activity of CLCC1 is required to maintain ER ion homeostasis and induce ER-phagy, as shown by the induction of ER-phagy upon transient expression of CLCC1^S263R^ (Fig. 2g), whose conductance is half that of wild-type CLCC1^35^. Taken together, these findings suggest that SARS-CoV-2 ORF3a hijacks CLCC1 to disrupt normal ER functions.

How SARS-CoV-2 ORF3a expression and CLCC1 deficiency cause ER-phagy and nucleophagy remains unclear. As a chloride channel, CLCC1 is primarily involved in maintaining ion homeostasis. On the basis of the results from experiments with our newly developed reporters, Tg-induced luminal Ca^2+^ depletion did not trigger ER-phagy or nucleophagy but activated the UPR and enhanced LC3-dependent autophagic flux (Supplementary Figs. S4, 6). Tg triggers the formation of the ER whorl, in which the membrane undergoes gradual degradation, in response to acute ER stress^59^. Thus, CLCC1 deficiency-induced phenotypes, including ER-phagy, nucleophagy, and LC3 flux, are unlikely to be directly caused by an acute reduction in [Ca^2+^]_ER_.

Instead, CLCC1 deficiency may share mechanistic features with Tm-induced ER stress, which disrupts ER protein folding by inhibiting glycosylation and subsequently causing ER-phagy and nucleophagy (Supplementary Fig. S6). We hypothesized that the loss of CLCC1 chloride channel activity impairs ER folding ability. Our study aligns with a previous report^35^ indicating the upregulation of UPR-related genes and 26S proteasome-related genes (Fig. 2b) and the observation of K48-specific ubiquitin signals (Fig. 7i), supporting the accumulation of unfolded and ubiquitinated proteins in cell models and mouse models. *CLCC1*-deficient cells (Fig. 2j) and mice^35^ are sensitive to Tm-induced ER stress, which links CLCC1 to ER protein folding ability.

CCPG1, an ER-phagy receptor, recognizes luminal cargo for degradation to manage ER stress^51,60^. In *CLCC1*-deficient cells, CCPG1 was highly expressed and further upregulated upon Tm treatment, linking CLCC1 to ER-phagy (Fig. 2j; Supplementary Fig. S5g). Recently, hepatic CLCC1 has been shown to interact with TMEM41B to regulate ER lipid metabolism^61,62^. Interestingly, TMEM41B has also been identified as an autophagy modulator^63,64^, which directly bridges CLCC1 to autophagy machines in most types of cells. Thus, CLCC1 deficiency may cause ER-phagy through reduced ER folding ability and luminal accumulation of unfolded proteins. Another explanation for ER-phagy caused by CLCC1 deficiency is that CLCC1 deficiency leads to ER membrane curvatures, such as swelling and fragmentation^35^, which can be sensed by the ER-phagy receptor FAM134B^65^. Therefore, ER-phagy and nucleophagy are activated in response to ER misfolded protein accumulation and/or ER membrane curvature caused by CLCC1 deficiency and SARS-CoV-2 ORF3a expression.

SARS-CoV-2 ORF3a has been previously reported to interact with the high mobility group box 1 (HMGB1)/Beclin 1 (BECN1) complex to trigger ER-phagy^66^. However, the HMGB1/BECN1 complex may also be recruited to SARS-CoV-2 ORF3a-enriched organelles, such as lysosomes and endosomes, which may limit the efficiency of ER-phagy. Thus, the SARS-CoV-2 ORF3a hijacks the host protein CLCC1 to cause global ER dysfunction and ER-phagy initiated by different receptors, providing a distinct explanation for ER-phagy activity.

The blebs observed in nuclear envelopes in SARS-CoV-2 ORF3a-expressing cells are striking. In recent works, the blebs in *CLCC1*-knockout cells were thought to be the consequence of the disruption of nuclear envelope fusion^61,67^. Experimental evidence is still lacking to clarify how the CLCC1 homomultimers facilitate nuclear pore complex assembly in vivo and in vitro. Here, we propose that the blebs are intermediates of nucleophagy and perinuclear ER-phagy. First, a reporter based on the nucleophagy receptor LMNB1 experimentally supported that CLCC1 deficiency causes nucleophagy (Fig. 2l). Second, endogenous LMNB1 expression was altered in *CLCC1*-deficient cells and SARS-CoV-2 ORF3a-expressing mouse brains, indicating the occurrence of nucleophagy (Figs. 2c, j and 7f). Third, the ER buds from the outer layer of nuclear envelopes evaded and enclosed the cytoplasm that seemed to form DMVs (Figs. 2a, 7g; Supplementary Video S1). DMV formation, especially in perinuclear regions, is a common feature of viral infections, including SARS-CoV-2^23^. Interestingly, TMEM41B is also required for DMV formation during coronavirus and pan-flavivirus infection^68–70^. It is possible that ORF3a–CLCC1 clusters, together with TMEM41B scramblase, change the curvature of the local membrane to form DMVs.

The topological similarity between SARS-CoV-2 ORF3a and CLCC1 is intriguing because they both possess three TMs, a luminal N-terminus, and a cytosolic C-terminus^9,10,35^. SARS-CoV-2 ORF3a exists mainly as a homodimer and then assembles into tetramers and oligomeric structures in vitro^9^, potentially recruiting CLCC1 to form ORF3a–CLCC1 clusters and blocking the conformational change of CLCC1 for chloride release. Alternatively, TM2 of ORF3a may be inserted into the CLCC1 complex, directly disrupting the chloride-permeable pathway. Future cryo-EM studies of the ORF3a–CLCC1 complex may elucidate these mechanisms.

Previous studies have shown that SARS-CoV-2 ORF3a interacts with VPS39 and inhibits autophagosome‒lysosome fusion, thereby blocking autophagic flux^15,16^. An alternative interpretation of our findings is therefore that the ER components observed in intracellular vesicles reflect autophagosome accumulation rather than ER-phagy. In this dose-dependent framework, different concentrations of ORF3a would compete for binding to ER-resident CLCC1 or lysosome-associated VPS39, and the functional outcome (ER-phagy vs flux blockade) would be determined by ORF3a abundance and binding affinity.

**Fig. 8:**
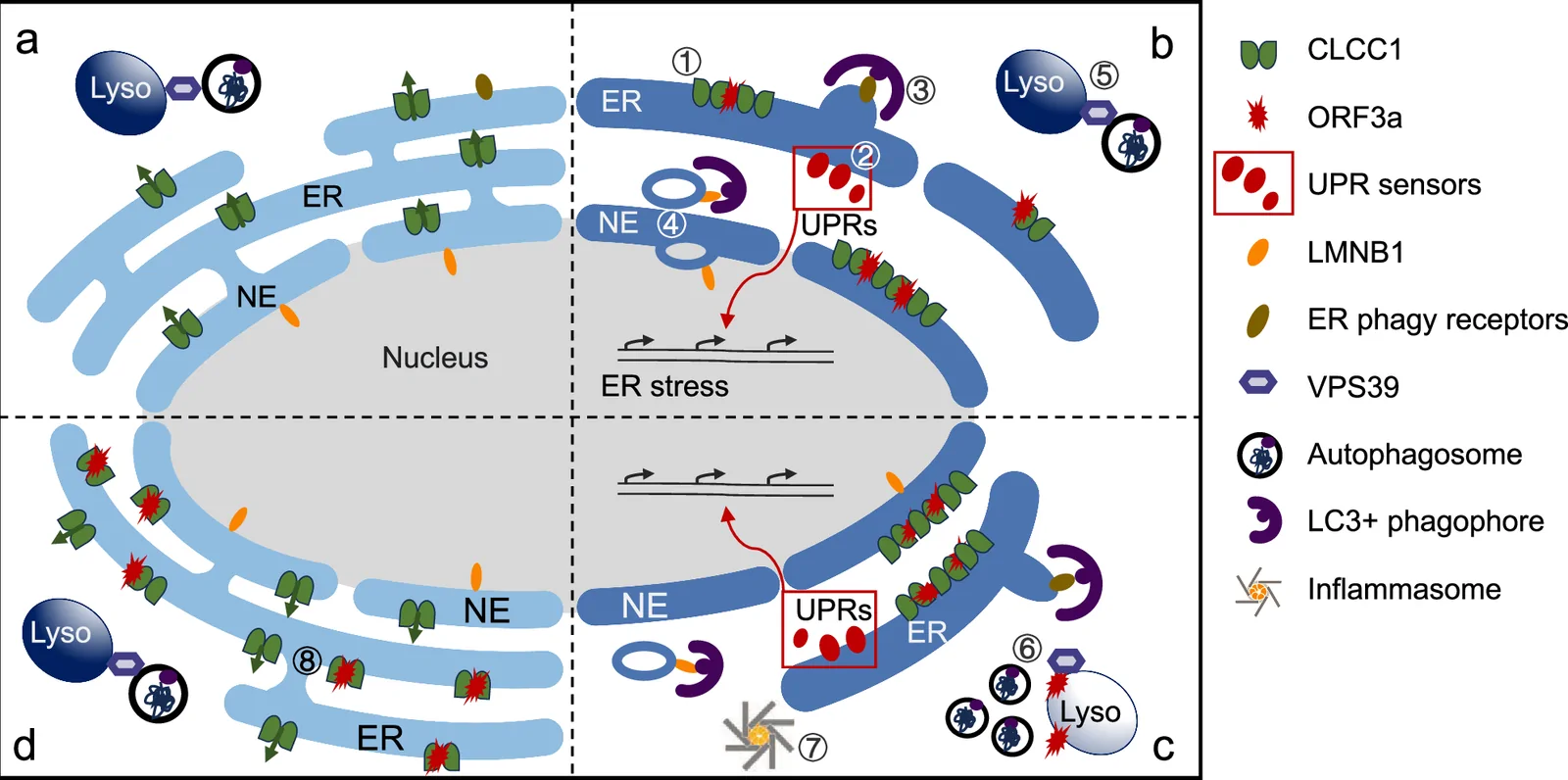
Illustration of the working model of the spatiotemporal functions of ORF3a in host cells and CLCC1 therapeutic potential as a host restriction factor. This model distinguishes early ER-localized ORF3a from late lysosome-associated ORF3a and explicitly separates ER-phagy initiation from late-stage autophagosome accumulation. Panel **a** shows the cells without SARS-CoV-2 infection (naïve condition). CLCC1, which is localized in the ER and outer nuclear envelope (NE), mediates chloride currents and controls ER ion homeostasis. Autophagy occurs at a basal level. Panel **b** depicts the early stage of SARS-CoV-2 infection. In this phase, ORF3a is expressed in the ER and binds to CLCC1. This binding impairs ER ion homeostasis (step 1) and triggers UPRs through three UPR sensors: IRE1, PERK, and ATF6 (step 2). The DMVs formed by viral NSPs and the expression of viral structural proteins reshape and burden the ER membrane. Consequently, ER-phagy and nucleophagy, triggered by ORF3a expression in the ER, remove the impaired ER membranes and nuclear membranes for material recycling. After lipidation, LC3 helps form phagophores and binds to ER-phagy receptors (step 3) or the nucleophagy receptor LMNB1 (step 4). Autophagy substrates, such as damaged membranes enclosed in autophagosomes, are eventually degraded by lysosomes (Lyso) (step 5). Panel **c** describes the late stage of SARS-CoV-2 infection. During this phase, the expression level of ORF3a gradually increases, saturating all CLCC1 proteins in the ER. Newly synthesized ORF3a subsequently escapes to lysosomes and inhibits autophagy in a VPS39-dependent manner, leading to the accumulation of autophagosomes (step 6). In virus-infected host cells, the immune response is triggered by inflammasome activation, which is characterized by caspase-1 cleavage (step 7). Panel **d** presents a therapeutic approach to address the toxicity caused by increasing CLCC1 expression (step 8). On the one hand, overexpression of CLCC1 directly restored ER function by re-establishing ion homeostasis through its channel activity. On the other hand, CLCC1 overexpression results in the retention of ORF3a in the ER, preventing the lysosomal targeting of ORF3a and indirectly benefiting the autophagy machinery.

While this scenario is conceptually plausible, several observations argue against a purely static competition model. First, our ER-phagy reporter functions as an irreversible molecular timer. The loss of pHluorin fluorescence reflects prior lysosomal acidification of ER membrane cargo and therefore records an earlier degradation event (Supplementary Fig. S6). This signal cannot be generated by autophagosome accumulation alone and is not reversed by subsequent lysosomal blockade. Second, the separation-of-function mutant ORF3a-Y160A is defective in VPS39 binding and is unable to inhibit autophagic flux, yet it robustly induces ER-phagy in our assay (Fig. 2g). This genetic uncoupling indicates that ER-phagy initiation does not depend on flux inhibition and can occur independently of VPS39-mediated function. Third, biochemical and imaging analyses demonstrated that ORF3a‒CLCC1 interactions occur predominantly in the ER, whereas ORF3a‒VPS39 interactions are enriched in lysosomal compartments, supporting spatially distinct functional states rather than cytosolic competition for a single ORF3a pool (Fig. 3e–i). Together, these findings are most consistent with a spatiotemporally ordered model (Fig. 8) rather than a simple abundance-dependent competition mechanism.

This model follows directly from the life cycle of ORF3a and established principles of membrane protein trafficking. As a viral transmembrane protein, ORF3a is synthesized and inserted into the ER membrane. At early stages of expression, the interaction of ORF3a with the ER-resident channel CLCC1 is retained within the ER compartment, where it perturbs ER ion homeostasis and initiates ER stress responses (Fig. 1). Under these conditions, ER whorl formation, ER-phagy, and nucleophagy (Fig. 2) are activated as adaptive mechanisms to remove damaged ER membranes and recycle materials (Fig. 8b). As infection progresses, ORF3a levels increase, and a portion of the protein is redistributed from the ER to lysosomal compartments. In this later phase, ORF3a engages VPS39 and disrupts HOPS complex function, thereby inhibiting autophagosome‒lysosome fusion (Figs. 3 and 8c). This sequential model reconciles previously reported autophagy inhibition with our findings of ER-specific autophagy induction and provides a unified mechanistic explanation for the stage-dependent functions of ORF3a.

Previous studies have reported that SARS-CoV ORF3a does not significantly perturb bulk autophagy, mainly on the basis of LC3 lipidation assays and tandem GFP-RFP-LC3 reporters^16^. However, these approaches measure bulk autophagic flux and lack sensitivity for detecting selective ER-phagy. To overcome this limitation, we used an ER-targeted tandem reporter and increased its dynamic range by replacing eGFP with super-ecliptic pHluorin, enabling the quantitative detection of ER-specific degradation (Supplementary Fig. S6). Using this optimized system, we found that both SARS-CoV-1 and SARS-CoV-2 ORF3a induce ER-phagy (Fig. 4f). Interestingly, SARS-CoV-1 ORF3a does not efficiently inhibit autophagosome‒lysosome fusion, whereas SARS-CoV-2 ORF3a uniquely engages VPS39 and blocks autolysosome formation^15,16^, which reconciles prior observations with our findings and supports a model in which the induction of ER dysfunction is a conserved ancestral function of ORF3a, whereas lysosomal flux inhibition is SARS-CoV-2-specific evolutionary acquisition.

In addition, the ER-associated functions of ORF3a proteins from related sarbecoviruses, including PCoV_GX and bat coronavirus HKU3, were similar in our assays (Fig. 4i–l). This conservation across phylogenetically distinct coronaviruses further supports the idea that ER-localized ORF3a activity is an evolutionarily conserved function, which raises intriguing questions regarding host–virus coevolution. Future studies are needed to determine how natural reservoir hosts tolerate or buffer ORF3a-mediated ER stress, potentially through adaptive modifications in ER homeostasis pathways or CLCC1-associated mechanisms.

Importantly, we demonstrated that overexpression of CLCC1 can rescue ORF3a-mediated toxicity and inhibit SARS-CoV-2 replication through the retention of ORF3a in the ER (Figs. 5 and 8), thereby providing a conceptual foundation for future therapeutic development.

AAV-PHP.eB-mediated expression of ORF3a in the mouse brain caused severe neurological impairment, including profound motor deficits and early lethality, accompanied by widespread neuropathology (Fig. 7). Similar motor and autophagy-related abnormalities have been reported in SARS-CoV-2 infection and ORF3a expression models^71–73^, supporting the physiological relevance of our observations. Importantly, ORF3a expression in vivo recapitulated key molecular features of CLCC1 dysfunction, including ER stress induction, dysregulated ER-phagy and nucleophagy, and the accumulation of ubiquitinated proteins (Fig. 7f–i). These mechanistically proximal phenotypes establish that the ORF3a–CLCC1 pathway, which was defined in vitro, also functions in the brain in vivo, linking ER homeostasis disruption to neurological consequences.

In summary, we elucidated the effects and mechanisms of the ORF3a–CLCC1 interaction in disrupting ER function and causing ER-phagy and nucleophagy, providing strong evidence that CLCC1–ORF3a could be a therapeutic target against SARS-CoV-2 infection. Further studies are warranted to validate the role of the ORF3a‒CLCC1 interaction in human patients and to help develop effective treatments. One potential strategy could be to deliver human CLCC1 to restore the functions of the ER and lysosome by targeting ORF3a of not only SARS-CoV-2 but also other beta-coronaviruses, which are still public health threats worldwide.

## Materials and methods

### Cell culture

The 293FT, U2OS, Caco-2-hACE2, and Vero cells were maintained in Dulbecco’s modified Eagle’s medium (DMEM; Corning, 10-013-CVR) supplemented with 10% heat-inactivated fetal bovine serum (FBS; Gemini, 900-108) and 1% penicillin/streptomycin (GE Healthcare). The plasmids were transfected into cells using Lipofectamine 3000 (Invitrogen, L3000015) following the manufacturer’s protocol for functional assays. Mycoplasma contamination was tested routinely by PCR (Forward primer: GGGAGCAAACAGGATTAGATACCCT; Reverse primer: TGCACCATCTGTCACTCTGTTAACCTC) and confirmed by Hoechst 33342 or Hoechst 33258 staining.

### Gene synthesis and molecular biology

Full-length CLCC1 (*Danio rerio* (XM_002667211.5), *Xenopus tropicalis* (XM_002932173.5), *Anolis carolinensis* (XM_003223596.3), and *Gallus gallus* (XM_422186.6)) was synthesized and subcloned into pIRES2-ZsGreen1 by Genewiz. The sequences of hLMNB1, hRAMP4, SARS-CoV-1 ORF3a, PCoV_GX ORF3a, and BtCoV HKU3 ORF3a were synthesized by Beijing Xianghong Biotechnology. In the FACS assays, exogenous genes for transient expression were subcloned into pIRES-miRFP670S. The pIRES-miRFP670S plasmid uses pcDNA3.1 as the backbone, which is inserted with an IRES and smaller but brighter miRFP670 (miRFP670S) after the multiple cloning sites. The truncation and mutation encoding gene fragments were generated by PCR, subcloned into the target plasmid using the Seamless Cloning Kit (Beyotime, D7010M), and validated by Sanger sequencing.

### Establishment of stable cell lines based on lentivirus infection

Lentiviruses were produced by co-transfecting 293FT cells with the transfer constructs, pMD2.G (Addgene, #12259) and psPAX2 (Addgene, #12260), by linear PEI (MW 25,000; Polysciences). The medium containing lentivirus was collected 48–72 h post-transfection, filtered to remove debris, and then added to fresh medium with an equal amount when the cells were plated for infection. To generate a stable cell line expressing human shRNA (MissionRNAi, Sigma), 293FT cells were selected with 2 g/mL puromycin (Gibco) after 48 h of infection and maintained with 1 µg/mL puromycin. To construct genetically encoded probes and reporters, we modified pLJM1-EGFP (Addgene, #19319) by replacing EGFP with our target sequences. After 48 h of 2 µg/mL puromycin-resistant selection, the cells expressing the probes and reporters were maintained in medium supplemented with 1 µg/mL puromycin and used within one week.

### Measurement of ER luminal [Cl^–^], [K^+^], and [Ca^2+^] concentrations

To measure the ER luminal ionic concentration at steady state, we expressed genes of interest in 239FT cells stably expressing a chloride sensor, a potassium sensor, and a ratiometric ER-localized calcium sensor, GCaMP6-150, fused with monomeric DsRed and KDEL sequences. After 24 h of transfection, the 293FT cells were digested and resuspended in DMEM (Gibco, 31053028) without phenol red for subsequent FACS recording.

### UPR reporter-related constructs

The UPR reporters were synthesized by Genewiz and subcloned into pGL4.29 after the cyclic AMP response element cassette, and luciferase gene between *Nhe*I and *Eco*RI was removed. Due to the splicing of *XBP1* upon IRE1 activation, the CMV promoter drives the expression of the XBP1 mini gene^74^ and GFP fusion protein. When PERK is activated, the translation of ATF4 may occur through a specific start codon^74^; otherwise, the translation of short nascent peptides stops quickly because of the premature stop codon. Thus, the 5′-UTR and N-terminal region of ATF4 fused with GFP were subcloned into pGL4.29 under the control of the CMV promoter. To monitor ATF6 translocation, the 5× ATF6 binding elements (modified from p5×ATF6-GL3; Addgene, #11976) were used to initiate GFP expression. The pGL4.29 plasmids harboring the UPR reporters were co-transfected into 293FT cells with the genes of interest, and the UPR signals were assessed by the proportion of GFP^+^ cells and mean GFP intensities.

### Western blotting and immunoprecipitation

For western blotting, the cultured cells or tissues were lysed in RIPA buffer (25 mM Tris-HCl, pH 7.4, 150 mM NaCl, 1% NP-40, 0.1% SDS, protease inhibitor cocktail, and PMSF). The cell debris was removed by centrifugation at 13,000× *g* for 5 min after incubation for 20 min on ice. The supernatant was boiled with 2× SDS loading buffer, and the proteins were separated on FuturePAGE precast gels (Ace, ET15412L) and transferred to a PVDF membrane (GE Healthcare) using fast-transfer buffer (NCM Biotech, WB4600). The blot was soaked in blocking buffer (PBS solution containing 0.1% Tween-20 and 5% skim milk) for 1 h at room temperature (RT) and incubated overnight with the primary antibody at 4 °C and then with the HRP-conjugated secondary antibody at RT for 60 min. After being probed with the appropriate antibodies, the chemiluminescence signals of the bands were captured by a Tanon 5200 Multi-system. The digital files were analyzed by ImageJ. The integrated density of the GAPDH band was divided by that of the target proteins from the same lane.

For immunoprecipitation (IP), the cultured cells or tissues were lysed in IP buffer (25 mM Tris-HCl, pH 7.4, 150 mM NaCl, 1% NP-40, protease inhibitor cocktail, and PMSF). The cell debris was removed by centrifugation at 100,000× *g* for 60 min to reduce unspecific binding from unsolved membranes. Anti-FLAG M2 magnetic beads (Millipore, M8823), FLAG nanobody-conjugated beads, and GFP nanobody-conjugated beads (Shenzhen KangTi Biopharma Technology, KTSM1338, KTSM1334) were used to capture the tagged proteins. The beads were pre-treated with lysis buffer and incubated with the supernatant of the cell lysate at 4 °C for at least 3 h or overnight. To immunoprecipitate the untagged proteins, the antibodies were prebound to Dynabeads (Invitrogen, 10014D) in lysis buffer at RT for 30 min, washed with lysis buffer three times, and then added to the supernatant and incubated at 4 °C for at least 3 h or overnight. The beads were subsequently washed five times with washing buffer (25 mM Tris-HCl, pH 7.4, 150 mM NaCl, 0.05% Tween-20, and protease inhibitor cocktail). The immunoprecipitated proteins were eluted directly with 2× SDS loading buffer at 95 °C for 5 min. To elute the FLAG-tagged proteins, washing buffer containing 500 ng/μL 3× FLAG peptide (Beyotime, P9801) was added to the beads and the mixture was rotated at 4 °C for 30 min. Then, the supernatants were collected, and 2× SDS loading buffer was added and boiled at 95 °C for 5 min.

### Proximity labeling

Human *CLCC1* and TurboID-encoding genes were subcloned into the pLJM1-EGFP plasmid for lentivirus generation. The 293FT cells stably expressing the CLCC1-TurboID fusion protein were transfected with FLAG-tagged SARS-CoV-2 ORF3a. Biotin was added to the medium at a final concentration of 200 μM at 37 °C for 15 min. The cells were then washed with ice-cold PBS at least three times to stop the labeling and the IP buffer described above was added. The biotinylated proteins were captured using M-280 streptavidin beads (Invitrogen, 11205D) and eluted with 2× SDS loading buffer containing 50 μM biotin at 95 °C for 5 min.

### Recombinant protein isolation and pull-down assay in vitro

The recombinant CLCC1 was isolated according to a previous procedure^35^. Briefly, the His_10_-tagged CLCC1 protein was expressed in High Five cells using a baculovirus expression system (Invitrogen) and purified with Ni-NTA resin (Qiagen), followed by size-exclusion chromatography (Superose 6 Increase, GE Healthcare) and elution in TBS buffer (50 mM Tris-HCl, pH 7.4, 150 mM NaCl) containing 0.025% n-dodecyl-β-D-maltopyranoside (DDM, Inalco) and 2 mM DTT.

In the pull-down assay, 293FT cells expressing GFP-tagged SARS-CoV-2 ORF3a and GFP were lysed in pull-down assay buffer (10 mM PBS, pH 7.4, 1% DDM, protease inhibitor cocktail, and PMSF). Then, the GFP nanobody-conjugated beads were used to capture SARS-CoV-2 ORF3a-GFP and GFP, followed by six washes with pull-down assay buffer (5 min each, on ice). The CLCC1-His protein was diluted in pull-down assay buffer and added to the tubes containing the GFP beads described above, followed by incubation on a rotator for 2 h at 4 °C. The beads were then washed five times with washing buffer (10 mM PBS, pH 7.4, 0.05% Tween-20, and protease inhibitor cocktail).

### Immunofluorescence and imaging

For cultured cell immunostaining, the cultured 293FT and U2OS cells were fixed with 4% (w/v) paraformaldehyde (PFA) for 10 min, permeabilized and blocked with blocking buffer (PBS supplemented with 0.1% saponin (Sigma, S7900), 3% BSA and 2% goat serum) for 1 h, stained with primary antibody in blocking buffer overnight at 4 °C, and then incubated with secondary antibody in blocking buffer for 1 h at RT. The slides were mounted with Fluoromount-G (Southern Biotech, 0100-01) containing Hoechst 33342 (Sigma, B2261). When saponin was used as the detergent, the cells were washed with PBS containing 0.1% saponin every time. To detect the nuclear protein LMNB1, the cells were permeabilized with PBS supplemented with 0.2% Triton X-100 at RT for 15 min, and washed with PBS.

The PFA-fixed paraffin-embedded sections were deparaffinized using the standard protocol for tissue immunostaining. The sections were boiled in sodium citrate buffer (10 mM sodium citrate, pH 6.0) and cooled to RT for antigen retrieval. After antigen retrieval, the sections were blocked with blocking buffer (25 mM Tris, pH 7.4, 150 mM NaCl, 0.3% Triton X-100, and 3% BSA) at RT for 1 h and stained with the primary and secondary antibodies in the blocking buffer described above. TUNEL staining was performed using the TUNEL Apoptosis Detection Kit (FITC) (Yeasen, 40306ES20) according to the manufacturer's instructions. Images were acquired using a Zeiss LSM 880 confocal microscope using an Airyscan 2 detector.

For live imaging, the cells were cultured in a 35 mm glass-bottom dish (D35-14-1.5GI), and the dish containing normal medium was fixed in a chamber for carbon dioxide supply during imaging. Images were acquired using a Zeiss LSM 880 confocal microscope with an Airyscan 2 detector.

### RNA-seq and data analysis

RNA was extracted from 293FT cells expressing GFP and SARS-CoV-2 ORF3a-GFP using TRIzol reagent (Thermo, 15596018), and total RNA was sequenced by Annoroad Gene Technology. The RNA-seq data were analyzed on hg38 with hisat2 and Stringtie, and DESeq2 was used to identify DEGs. Genes with adjusted *P*-value < 0.05 were identified as DEGs. Enrichment analysis of the upregulated and downregulated genes was performed for the KEGG pathways. Pathways with adjusted *P*-value < 0.05 were identified as differentially expressed pathways.

### SARS-CoV-2 infection

The clinically isolated SARS-CoV-2 strain, C-Tan-nCoV Wuhan strain 01 (SARS-CoV-2 strain BetaCoV/Wuhan/IVDC-HB-01/2019 (C-Tan-HB01, GISAID accession no. EPI_ISL_402119)), was isolated from a human patient^75^. The virus was harvested, and the viral titer was determined by a micro-cytopathogenic efficiency assay in Vero cells. The Vero cells were seeded on Lab-Tek chamber slides, and the SARS-CoV-2 virus was added to the medium. After a 48 h infection, 4% PFA (w/v) in PBS was used to fix the cells and inactivate the virus. For the Western blot, the Vero cells were seeded on a six-well plate, and the SARS-CoV-2 virus was added to the medium. RIPA buffer containing protease inhibitors was added to the cells 48 h post-infection. The cell lysate supernatant was collected and subjected to western blotting after centrifugation at a maximum speed of 4 °C. Cells without added virus were used as a control. All experiments involving SARS-CoV-2 were performed in biosafety level 3 (BSL) laboratories at the National Institute for Viral Disease Control and Prevention, China CDC.

### Virus amplification in BSL-2 containment

Vero cells were grown to 80%–90% confluency in T-25, and HSV-1-GFP (Herpes simplex virus 1-GFP, Kos strain) virus stock was subsequently added to the culture medium at an MOI equal to 1 and incubated for 48 h. The supernatant was collected when 80% cytopathic effect was observed and stored at –80 °C before use. For SARS-CoV-2 ΔN/GFP trVLPs amplification, the host cell was infected with lentivirus, which delivered the N gene and mCherry cassette, and was subsequently sorted by FACS. The virus stock was added to the culture medium at an MOI equal to 0.1 and incubated for 72 h. The supernatant was collected when 80% cytopathic effect was observed and stored at –80 °C before use. The infectious titers of the supernatant were determined by a TCID_50_ endpoint dilution assay using the Reed–Muench method.

### High-content imaging analysis

Caco-2-hACE2 cells infected with SARS-CoV-2 ΔN/GFP trVLPs were cultured in 96-well plates and imaged on the Operetta High-Content Imaging System (CLS) (Revvity, USA), supplying 5% CO_2_ at 37 °C. Images from 16 fields per well were taken with a 10× Air/NA0.3 objective using the laser non-confocal method on two channels, bright field (exposure: 3 ms, power: 8%) and FITC (exposure: 50 ms, power: 50%), and then analyzed by the Operetta CLS.

### Stable cell line using the Tet-on 3 G inducible PiggyBac system

The plasmid HP138-puro (Addgene, #134246) harboring GFP-FAM134B was digested using *Nhe*I and *Age*I, and the gene cassettes of GFP, ORF3a-GFP, ER-ORF3a, and CLCC1-GFP were inserted, respectively. The plasmids of PB-CAG-BGHpA (Addgene, #92161) and HP138-puro were co-transfected into the cells at a ratio of 2:1. The culture medium containing puromycin was used for selection (2 μg/mL) 24 h post-transfection and maintenance (1 μg/mL). To induce exogenous genes, 1 μg/mL doxycycline (Sigma, D5207) was added to the culture medium.

### Antibodies

The following primary antibodies were used, including anti-CLCC1 (1:10,000 in western blot, 1:1000 in immunofluorescence, 2 μg/sample in IP, made in house), anti-FLAG (1:6000, western blot, Abmart, M20008L), anti-GAPDH-HRP (1:10,000, western blot, Proteintech, 60004), anti-Tubulin-HRP (1:10,000, western blot, Proteintech, 66031), anti-ORF3a (1:5000, western blot, 1:200, immunofluorescence, ABclonal, A20234), anti-His (1:1000, western blot, Cell Signaling Technology, 2365), anti-HA (1:5000, western blot, 1:1000, immunofluorescence, Abmart, M20003L), anti-LMNB1 (1:8000, western blot, 1:1000, immunofluorescence, Proteintech), anti-Bip (1:300, immunofluorescence, Abcam), anti-ubiquitin (1:200, immunofluorescence, Cell Signaling Technology, 3936), anti-ubiquitin-K48 (1:200, immunofluorescence, Abcam, ab140601), anti-VPS39 (2 μg/sample in IP, Proteintech, 16219-1-AP), anti-ATL3 (1:8000, western blot, Proteintech, 16921-1-AP), anti-FAM134B (1:6000, western blot, Proteintech, 21537-1-AP), anti-RTN3 (1:8000, western blot, Proteintech, 12055-2-AP), anti-CCPG1 (1:2000, western blot, Proteintech, 13861-1-AP), anti-SEC62 (1:6000, western blot, Proteintech, 28693-1-AP), anti-TEX264 (1:4000, western blot, Proteintech, 25858-1-AP), anti-NUP98 (1:6000, western blot, Abcam, ab50610), anti-LC3 (1:1000, western blot, MBL, PM036), anti-LC3A/B (1:1000, western blot, Cell Signaling Technology, 4108), anti-GFP (1:5000, western blot, 1:1000, immunofluorescence, Abcam, ab6556 and Immunoway, YM3124), anti-caspase-3 (1:3000, western blot, Cell Signaling Technology, 14220), anti-mCherry (1:5000, western blot, Abcam, ab167453), anti-caspase-1 (1:10,000, western blot, Proteintech, 22915-1-AP), anti-Iba1 (1:1000, immunofluorescence, ABclonal, A19776), and anti-GFAP (1:1000, immunofluorescence, Cell Signaling Technology, 3670), anti-LAMP1 (1:1000, immunofluorescence, Cell Signaling Technology, 9091 and 15665). For secondary antibodies, we used Alexa-conjugated secondary (488, 555, 647) antibodies (Life Technologies; Molecular Probes) at 1:500, and HRP-linked secondary antibodies (GE Healthcare) at 1:6000.

### CRISPR/Cas9-based gene knockout

The dual gRNAs targeting the human SEC62 gene (sequence inserted: CACCAATATGATGGGTCACCGG and AAAGCAGTGGACTGTCTTTTGG) were subcloned into pX458 (Addgene, #48138) to improve the knockout efficiency. These two gRNAs are driven by the U6 promoter and H1 promoter, respectively. After 24 h of transfection, the 293FT cells were digested, suspended in PBS, and subjected to flow cytometry (BD Influx). The GFP^+^ cells were subsequently sorted for subcloning. The SEC62 antibody was confirmed by western blotting using single clones of the SEC62-KO cells.

### Lentivirus-based shRNA transduction

To validate the antibodies, we conducted gene knockdown experiments using shRNA targeting our genes of interest. The knockdown efficiency was assessed by western blotting, with cells expressing scrambled shRNA serving as the control. The sequences of shRNAs (MissionRNAi, Sigma) targeting human genes are listed below.

*CLCC1*, CCGGTTCATGTGCTGAGACATATAGCTCGAGCTATATGTCTCAGCACATGAATTTTTG; *ATL3*, CCGGCCCTTGCAGATGGCTCTTAATCTCGAGATTAAGAGCCATCTGCAAGGGTTTTTG; *FAM134B*, CCGGCCACAGACAGACACTTCTGATCTCGAGATCAGAAGTGTCTGTCTGTGGTTTTT; *CCPG1*, CCGGTACCCGAAGACAGTATCTATACTCGAGTATAGATACTGTCTTCGGGTATTTTTG; *NUP98*, CCGGCCCTTGCAGATGGCTCTTAATCTCGAGATTAAGAGCCATCTGCAAGGGTTTTTG; *RTN3*, CCGGCGAGATCAGACCAAGTCAATTCTCGAGAATTGACTTGGTCTGATCTCGTTTTTTG; *TEX264*, CCGGCCAGGAAGACCAGATCCATTTCTCGAGAAATGGATCTGGTCTTCCTGGTTTTTTG.

### Development of novel reporters for ER-phagy, nucleophagy, and autophagic flux (RaMETR, RaMNuTR, and RaMLAFR)

The ratiometric reporters were developed with the same strategies: mCherry as an internal control and pHluorin to monitor changes in the surrounding pH. The ER-phagy substrate (ER-resident hRAMP4), nucleophagy receptor (hLMNB1), and hLC3B were fused to the C-terminus of pHluorin, respectively. The gene cassettes encoding these elements were subcloned into pLJM1-EGFP for lentivirus packaging, in which the EGFP cassette was removed. The 293FT cells were digested and resuspended for recording by FACS after 24 h of transfection. To characterize these reporters, the following reagents were used: EBSS (Invitrogen, 24010043), Torin 1 (Selleck, S2827), tunicamycin (Millipore, 654380), BFA (Selleck, S7046), thapsigargin (Sigma, T9033), and BafA1 (Selleck, S1413).

### Flow cytometry

An LSRFortessa flow cytometer (BD Biosciences) was used to record the fluorescent signals, and Aria Fusion SORP (BD Biosciences) was used to sort the cells. For the GFP, Cl^–^ sensor, K^+^ sensor, ER-GCaMP6-150, and pHluorin, we used the FITC channel (488 nm); for DsRed and mCherry, we used the PE channel (561 nm); for miRFP670S, we used the APC channel (647 nm); and for BFP, we used the Pacific Blue channel (405 nm). The cells were treated with DAPI (Beyotime) using the Pacific Blue channel (405 nm) to exclude dead cells. The data were analyzed using FlowJo X.

### TEM

The 293FT cells were seeded and cultured in 35 mm dishes and fixed with fixation solution (FS, 4% PFA (w/v) in 0.1 M phosphate buffer (PB, pH 7.4)) containing 2.5% glutaraldehyde at 37 °C for 30 min. Mice were perfused with 0.1 M PB at RT and then fixed with FS containing 2.5% glutaraldehyde in FS at 4 °C overnight. Similar regions of the olfactory bulb and hippocampus were cut into 200 μm pieces for embedding. Cell and tissue embedding and subsequent steps were performed at the Cell Biology Facility, Center of Biomedical Analysis, Tsinghua University. Briefly, the samples were fixed with 1% osmium tetroxide and 1.5% tetrapotassium hexacyanoferrate trihydrate for 1 h at 23 °C, followed by dehydration in a series of ethanol solutions (50%, 70%, 80%, 90%, and 100%) four times for 10 min each time. Infiltration was performed with 1,2-epoxypropane twice for 10 min each, followed by gradual infiltration with a mixture of 1,2-epoxypropane and Epon 812 resin for 8 h (SPI, America). The samples were subsequently infiltrated twice with pure Epon 812 resin and polymerized at 60 °C. Polymerized blocks were cut into ultra-thin sections (80 nm) using a Leica EM UC7 ultramicrotome (Wetzlar, Germany). Ultra-thin sections were mounted and dried on coated copper grids and stained on-grid with 2% uranyl acetate for 20 min, followed by lead citrate for 5 min. The stained sections were imaged using a Hitachi H-7650B and H-7800 transmission electron microscope (Tokyo, Japan).

### APEX2 labeling

The 293FT cells expressing APEX2-tagged protein were cultured in 35 mm dishes and treated with a fresh medium mixture with prewarmed fixation solution (0.1 M PB, pH 7.4) containing 2.5% glutaraldehyde at 37 °C for 5 min. The cells were fixed with fresh fixation solution (0.1 M PB) containing 2.5% glutaraldehyde at 37 °C for 10 min and transferred to ice for an additional 30 min. All subsequent steps were kept on ice. The cells were rinsed three times with 0.1 M PB for 5 min each time and incubated with 1.4 mM fresh DAB solution (Sigma, D5637) for 20 min, and then the solution was changed to 1.4 mM fresh DAB solution containing 0.03% H_2_O_2_ for almost 15 min (when most of the cells turned brown). To stop the reaction, the cells were rinsed three times with 0.1 M PB for 5 min each time. Cell embedding and subsequent steps were performed at the Cell Biology Facility at Tsinghua University, following the description of TEM above.

### Focused ion beam scanning electron microscopy (FIB-SEM) and 3D reconstruction

For SEM, the image stacks were acquired with 5 nm *x*–*y* pixels using a Zeiss Crossbeam 550 (Germany) with a 500 pA electron beam at 2 kV for imaging and a 3 nA gallium ion beam at 30 kV for FIB milling. Post-processing and analysis were performed in ORS Dragonfly software (v2022.2). All the image stacks were aligned and exported as a movie. To enhance the contrast, contrast-limited adaptive histogram equalization was used.

### AAV-PHP.eB administration and mice

AAV-PHP.eB virus harboring a GFP and SARS-CoV-2 ORF3a cassette (1 × 10^13^ vg/mL) was generated by Packgene Biotech and diluted to a final concentration of 50 × 10^10^ vg/600 µL in sterile PBS. At P22, the mice were anesthetized with isoflurane and received AAV via retro-orbital injection on both sides (1 × 10^11^ vg/20 g mice) as quickly as possible. After the injection, the eyes showed no apparent bleeding. Three to 4 weeks later, the mice were transcardially perfused with PBS after anesthetization and fixed with 4% PFA in PBS, followed by overnight fixation. The tissues were then prepared for embedding in paraffin blocks. C57BL/6 mice were purchased from the Laboratory Animal Resources Center at Tsinghua University and housed in a specific-pathogen-free animal facility. The mice were maintained on a 12/12-h light/dark cycle, 22–26 °C, with sterile pellet food and water ad libitum. The animal facility at Tsinghua University has been accredited by the China National Accreditation Service for Conformity Assessment (CNAS) since 2022. All animal protocols were approved by the Institutional Animal Care and Use Committee (IACUC) at Tsinghua University on the basis of the Guide for the Care and Use of Laboratory Animals (Eighth Edition, NHR).

## Supplementary information

Supplementary information

Supplementary Video S1

Supplementary Video S2

Supplementary Table S1

Supplementary Table S2
