# Supplementary material for: The accessory protein ORF3a hijacks the CLCC1 chloride channel to disrupt ER homeostasis in betacoronavirus pathogenesis
Source: Cell Discov. 2026 Sep 15;12:66. doi: 10.1038/s41421-026-00918-0 (PMC13575168; doi:10.1038/s41421-026-00918-0)
Supplement: Supplementary file 1 [file 41421_2026_918_MOESM1_ESM.pdf]

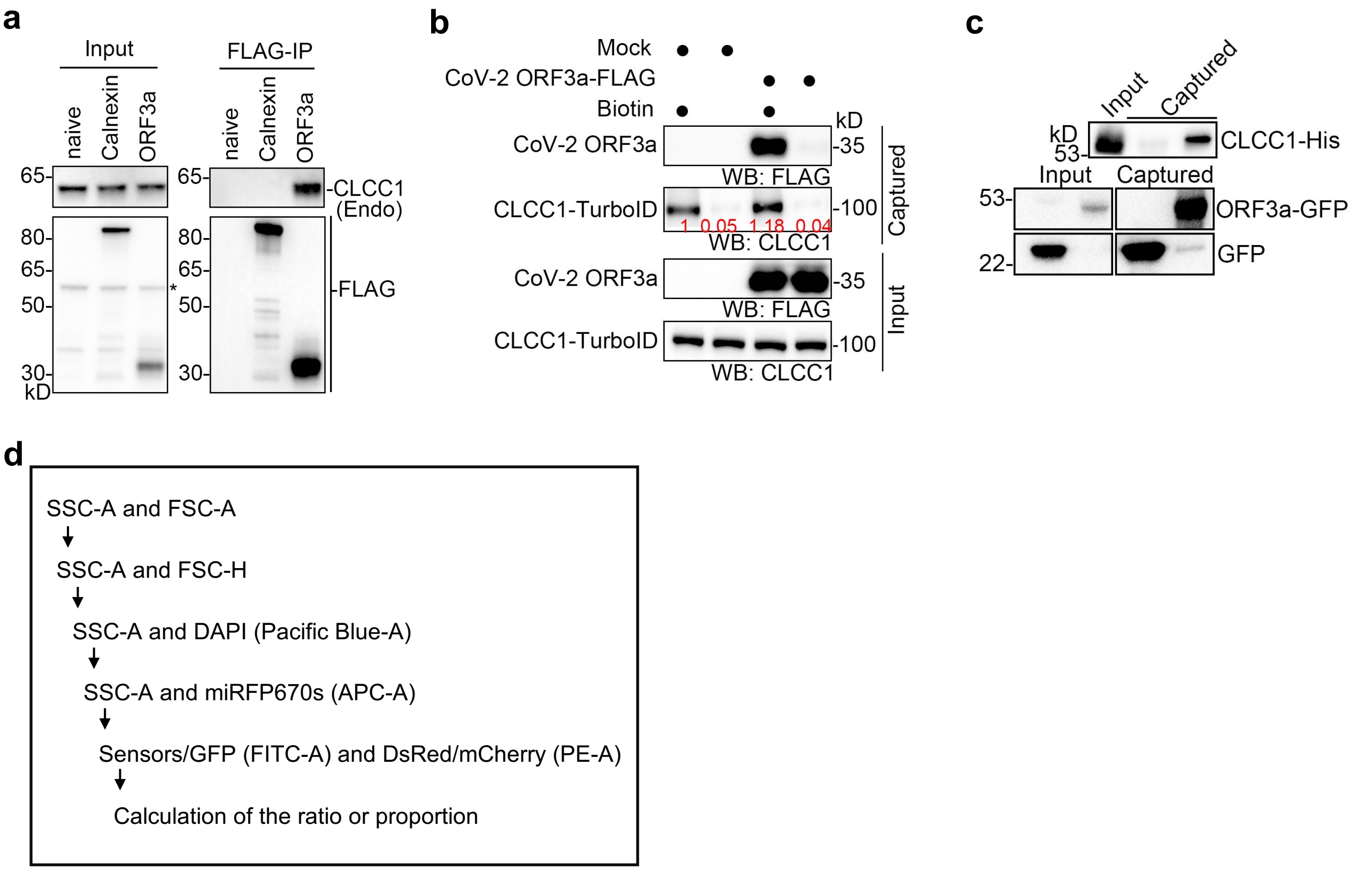

Supplementary Fig. S1 SARS-CoV-2 ORF3a interaction with CLCC1 *in vitro* and the gating strategies in flow cytometry.

a, SARS-CoV-2 ORF3a interacts with endogenous CLCC1. FLAG-tagged SARS-CoV-2 ORF3a was transiently expressed in 293FT, immunoprecipitated using anti-FLAG nanobody beads, and analyzed by western blot. FLAG-tagged Calnexin was used as a negative control.

b, SARS-CoV-2 ORF3a interacts with exogenous CLCC1. SARS-CoV-2 ORF3a was transiently expressed in 293FT cells expressing human CLCC1 fused with TurboID. The cells were treated with 200 μM biotin for 15 minutes. The biotinylated proteins were captured using streptavidin beads and applied to the western blot. The antibodies against CLCC1 and FLAG were used to detect the CLCC1 and SARS-CoV-2 ORF3a, respectively.

c, In vitro pull-down assay. GFP-tagged SARS-CoV-2 ORF3a expressed in 293FT cells was captured using GFP nanobody-conjugated beads and incubated with purified His-tagged CLCC1 protein. Bound proteins were analyzed by immunoblotting using anti-His and anti-GFP antibodies to detect CLCC1 and ORF3a, respectively. GFP alone served as a negative control.

d, The gating strategy of FACS assays.

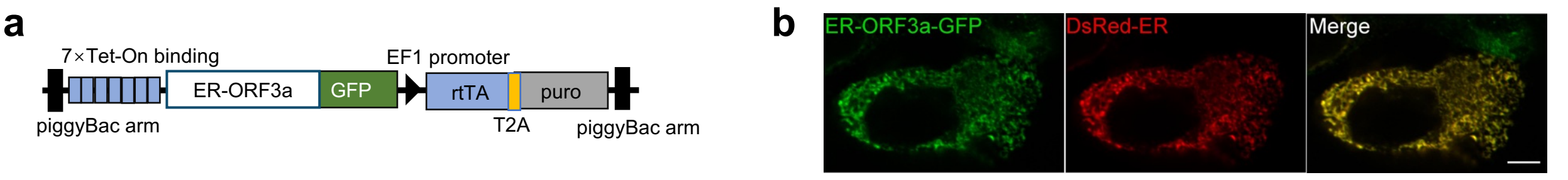

Supplementary Fig. S2 ER localization of ER-ORF3a-GFP.

a, Design of inducible expression of GFP-tagged ER-ORF3a.

b, The signal peptide of calreticulin facilitates the ER localization of ORF3a. The expression of ER-ORF3a-GFP was induced by doxycycline (Dox, 1 μg/ml) overnight. The DsRed2-ER (Takara, cat. 632409) was transiently expressed to label the ER in the fixed cells. Scale bar, 5 μm.

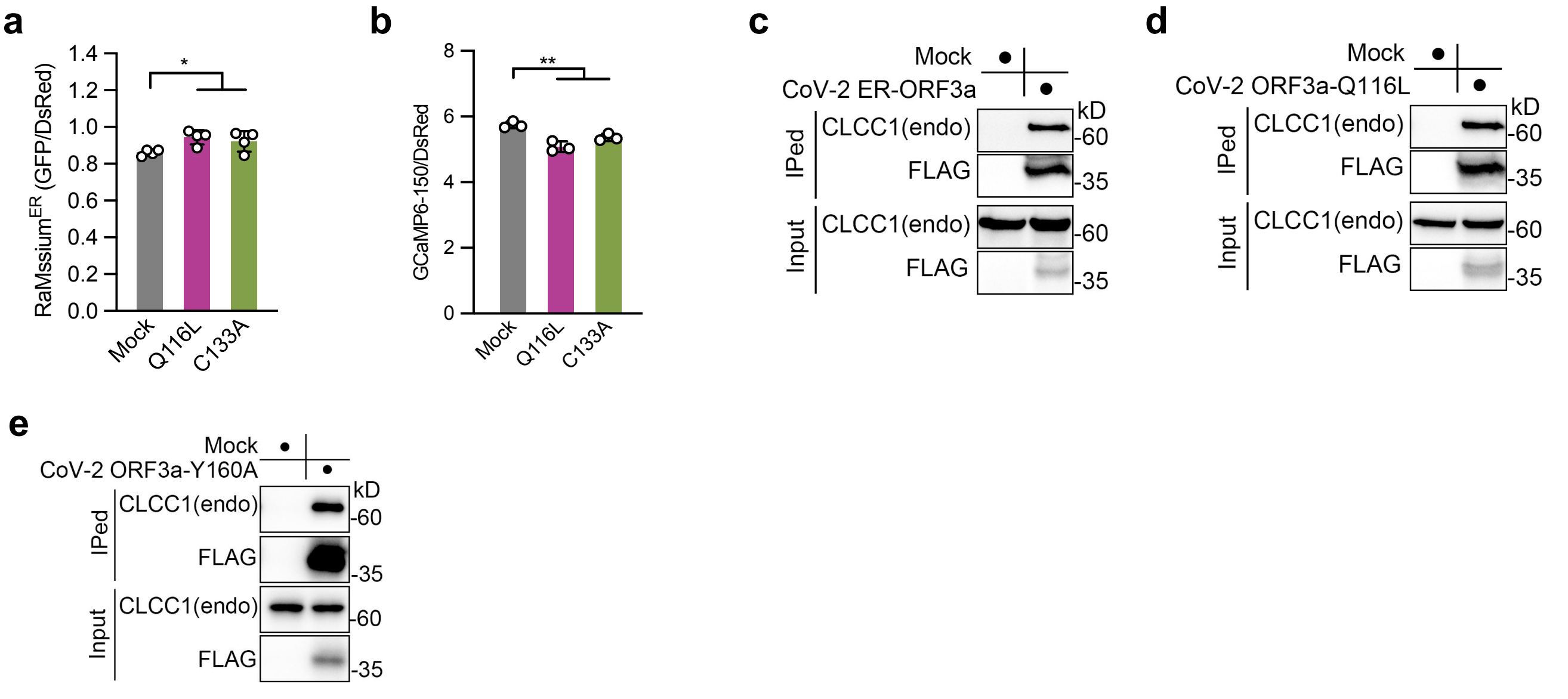

Supplementary Fig. S3 SARS-CoV-2 ORF3a mutants impair the ER ion homeostasis and interact with endogenous CLCC1.

a-b, The impairment of $[K^+]_{ER}$ and $[Ca^{2+}]_{ER}$ by SARS-CoV-2 ORF3a mutants.

c-e, Engineered SARS-CoV-2 ORF3a and its mutants interact with endogenous CLCC1. The FLAG-tagged mutant ORF3a proteins were transiently expressed in 293FT cells, immunoprecipitated by anti-FLAG M2 beads, and applied to western blot. The antibodies against CLCC1 and FLAG detected CLCC1 and SARS-CoV-2 ORF3a mutants, respectively.

Values are presented as mean ± SD. n.s., no significant difference; $^*P < 0.05$, by one-way ANOVA. In **a** and **b**, > 3000 miRFP670s+ cells were recorded per experiment, with $n \geq 3$.

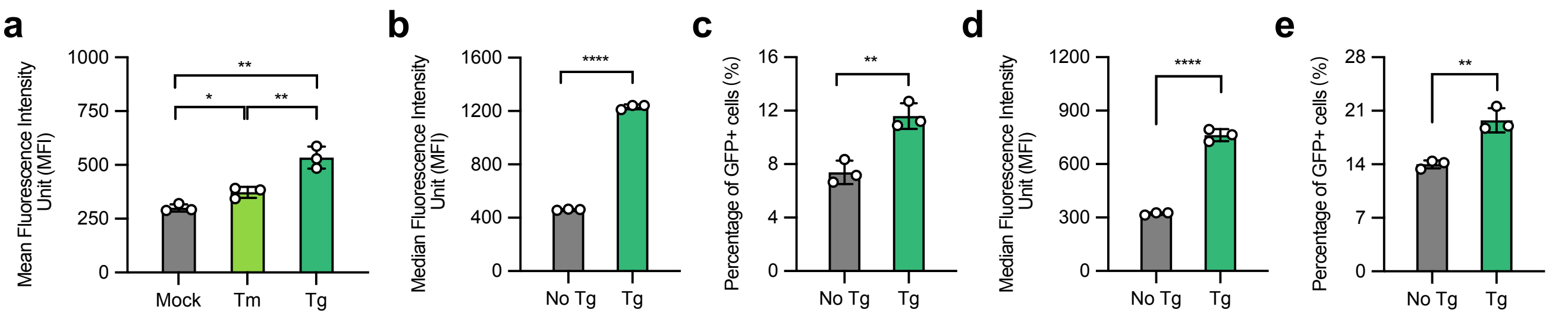

Supplementary Fig. S4 Validation of UPR reporters in 293FT cells by treatments with thapsigargin and tunicamycin.

a, Treatments with thapsigargin (Tg) and tunicamycin (Tm) induce *XBP1* splicing.

b-c, Tg treatment enhances ATF4 translation.

d-e, Tg treatment triggers ATF6 cleavage.

The 293FT cells transiently expressing UPR reporters were treated with Tg (500 nM) and Tm (1 μg/ml) overnight. The medium or mean intensity of GFP and the percentage of GFP+ cells among live cells (DAPI-) were measured. (**a**), approximately 1000 miRFP670s+ cells were recorded, and the mean intensity of GFP was used; (**b-e**), > 3000 miRFP670s+ cells were recorded per experiment, with n = 3. Values are presented as mean ± SD. *P < 0.05, **P < 0.01, ***P < 0.001, ****P < 0.0001, by student's *t*-test.

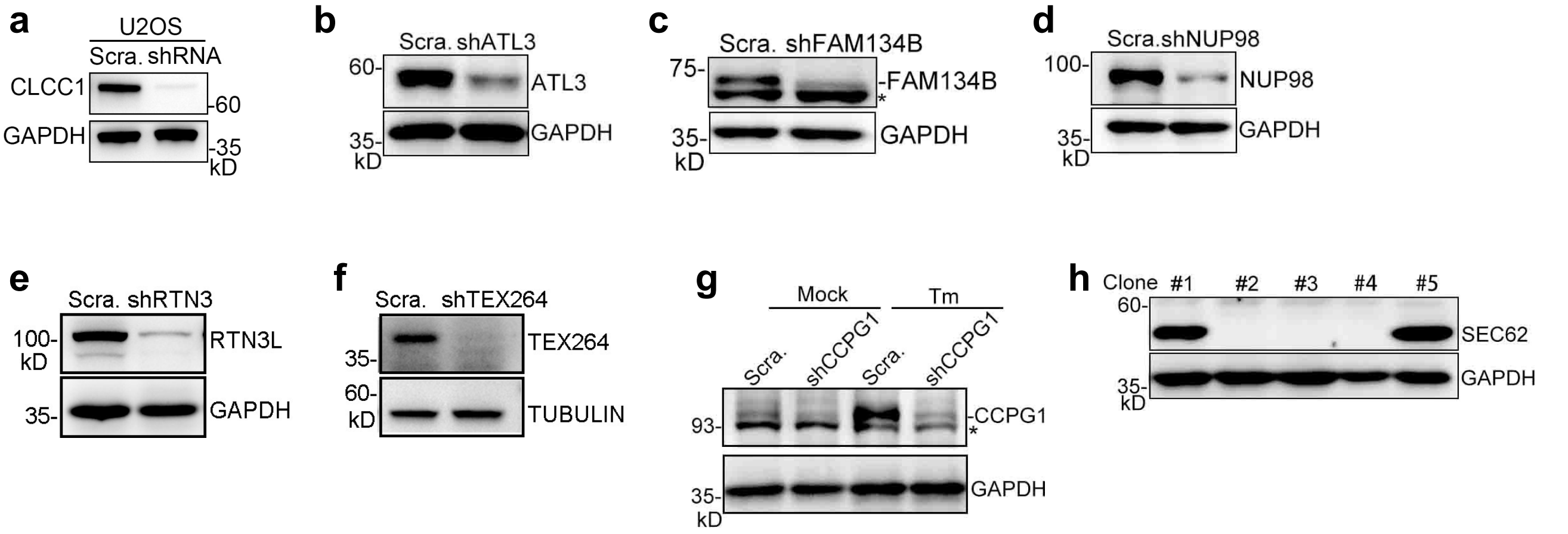

Supplementary Fig. S5 Validations of antibodies.

a, Validation of antibody against endogenous CLCC1. Western blot analysis of cell lysates from 293FT cells expressing either *CLCC1*-targeting shRNA (H3) or scrambled (Scra.) control, probed with CLCC1 antibody.

b-f, Validation of antibodies against endogenous ATL3, FAM134B, NUP98, RTN3L (long isoform of RTN3 is ER phagy receptor) and TEX264 using shRNA or scrambled control. Western blot analysis was performed to confirm the specificity of each antibody.

g, Validation of antibody against endogenous CCPG1. Treatment with Tunicamycin (Tm, 1 μg/ml for 18 hours) significantly increased CCPG1 expression. A western blot was performed for cell lysates from cells expressing *CCPG1*-targeting shRNA or scrambled (Scra.) control, probed with CCPG1 antibody.

h, Validation of antibody against endogenous SEC62. CRISPR/Cas9-based gene knockout was performed, and the cell lysates of subclones were applied to the western blot and probed with the SEC62 antibody.

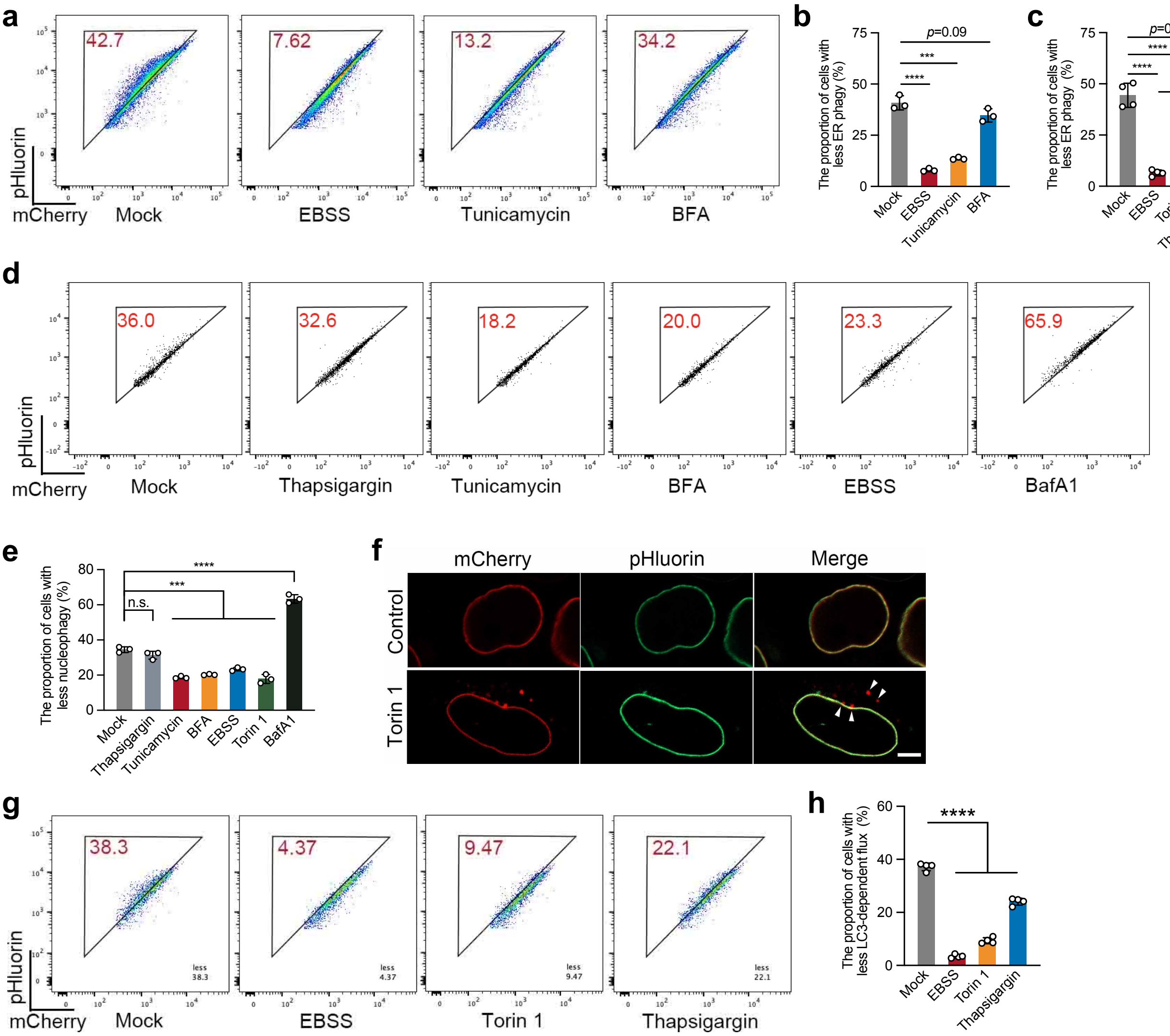

Supplementary Fig. S6

Supplementary Fig. S6 Validations of RaMETR, RaMNuTR, and RaMLAFR.

a-c, RaMETR characterization by FACS. 293FT cells stably expressing RaMETR were treated overnight with EBSS, Torin 1, tunicamycin (Tm), BFA, or thapsigargin (Tg). The live cells (DAPI-) were recorded by FACS. In mock conditions, approximately 40% of cells were defined as normal. During ER phagy, the proportion of cells with less ER phagy (brighter pHluorin fluorescence) decreases in the triangular gate.

d-e, RaMNuTR characterization by FACS. 293FT cells stably expressing RaMNuTR were treated with Tg, Tm, BFA, EBSS, Torin 1, or BafA1 (Bafilomycin A1) overnight.

f, Live imaging of RaMNuTR. U2OS cells stably expressing RaMNuTR were treated with Torin 1 (2 μM) for 4 hours before imaging. The mCherry puncta without pHluorin signal in the nuclear envelope and the cytoplasm indicate the nucleophagy triggered by Torin 1. Scale bar, 5 μm.

g-h, RaMLAFR characterization by FACS. 293FT cells stably expressing RaMLAFR were treated with EBSS, Torin 1, or Tg overnight.

The chemicals used in the FACS assays include Torin 1 (1 μM), Tm (1 μg/ml), Tg (500 nM), BFA (5 μg/ml), and BafA1 (200 nM). At least 1000 live cells were recorded per experiment, with $n \geq 3$. Values are presented as mean ± SD. n.s., no significant difference; *$P < 0.05$, **$P < 0.01$, ***$P < 0.001$, ****$P < 0.0001$, by student's *t*-test.

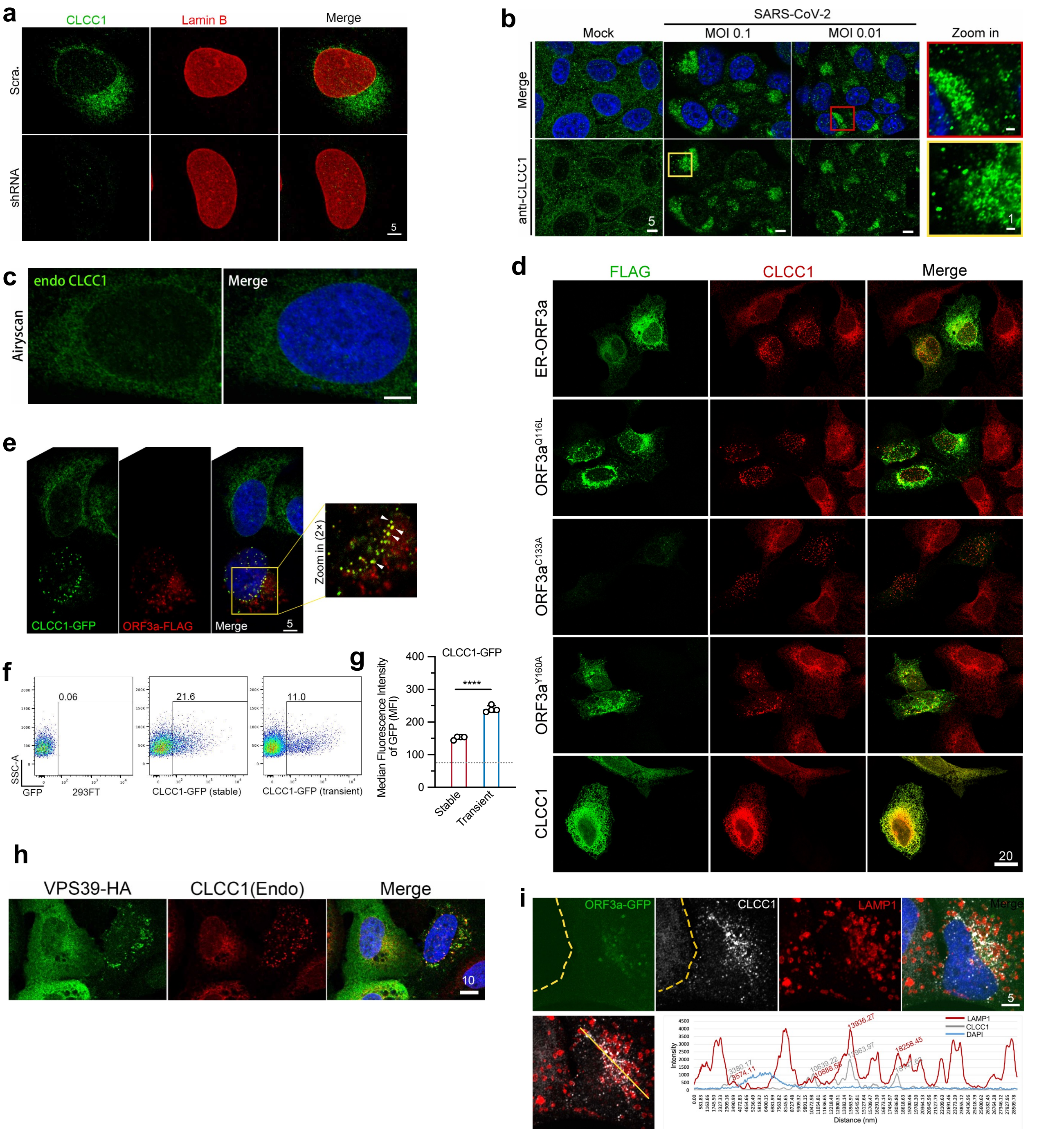

Supplementary Fig. S7

Supplementary Fig. S7 SARS-CoV-2 ORF3a changes CLCC1 expression patterns.

a, Validation of antibody against endogenous CLCC1. The fixed U2OS cells expressing *CLCC1*-targeting shRNA (H3) or scrambled (Scra.) shRNA were stained with CLCC1 antibody. Lamin B1 served as an internal control.

b, Airyscan imaging of endogenous CLCC1 in U2OS cells. CLCC1 was stained with the CLCC1 antibody.

c, Immunofluorescence analysis of CLCC1 in Vero cells infected with the SARS-CoV-2 virus at the indicated MOIs. Vero cells without virus infection served as the negative control.

d, Confocal imaging of exogenous CLCC1-GFP and FLAG-tagged SARS-CoV-2 ORF3a in U2OS cells. CLCC1 puncta were observed in U2OS cells stably expressing CLCC1-GFP when FLAG-tagged SARS-CoV-2 ORF3a was transiently expressed, but not the neighboring control cells. The co-localization of CLCC1 and SARS-CoV-2 ORF3a is labeled with white arrows. CLCC1 and SARS-CoV-2 ORF3a were detected by GFP and FLAG antibodies, respectively.

e, Confocal imaging of CLCC1 and FLAG-tagged SARS-CoV-2 ORF3a and its mutants in U2OS cells. FLAG-tagged SARS-CoV-2 ORF3a and its mutants and FLAG-tagged CLCC1 were transiently expressed and stained with FLAG antibody.

f-g, CLCC1-GFP expression levels in the transfected and stable cell line. (f)The CLCC1-GFP gene cassettes driven by the CMV promoter were either transduced via lentivirus or transfected into 293FT cells (middle and right panels). GFP signals were recorded by FACS. More than 2000 GFP+ cells were recorded per experiment. The naïve 293FT served as a negative control for gating. (g) Quantitative analysis of CLCC1 expression levels in 293FT cells stably or transiently expressing CLCC1-GFP in (f). The gay dashed line indicates the fluorescence background of the naïve 293FT cells in FACS. Values are presented as mean $\pm$ SD in (g). ****$P < 0.0001$, by student's *t*-test, with $n = 4$.

h, Immunofluorescence analysis of VPS39 and CLCC1. ORF3a was transiently expressed in U2OS cells expressing HA-tagged human VPS39. Endogenous CLCC1 and VPS39 were stained with the antibodies against CLCC1 and HA, respectively.

i, Immunofluorescence analysis of LAMP1 and CLCC1 puncta. GFP-tagged ORF3a was transiently expressed in U2OS cells. Endogenous CLCC1 and LAMP1 were immunostained with the antibodies against CLCC1 and LAMP1, respectively. The lower panel shows line-scan analysis of fluorescence intensity profiles for LAMP1 and CLCC1 puncta. Fluorescence intensities are plotted against distance, with peak positions of CLCC1 puncta and LAMP1 signals indicated.

Scale bar in **a-c**, **e**, **i**, 5 μm; in **d**, 20 μm; in **h**, 10 μm; in enlarged **b**, 1 μm.

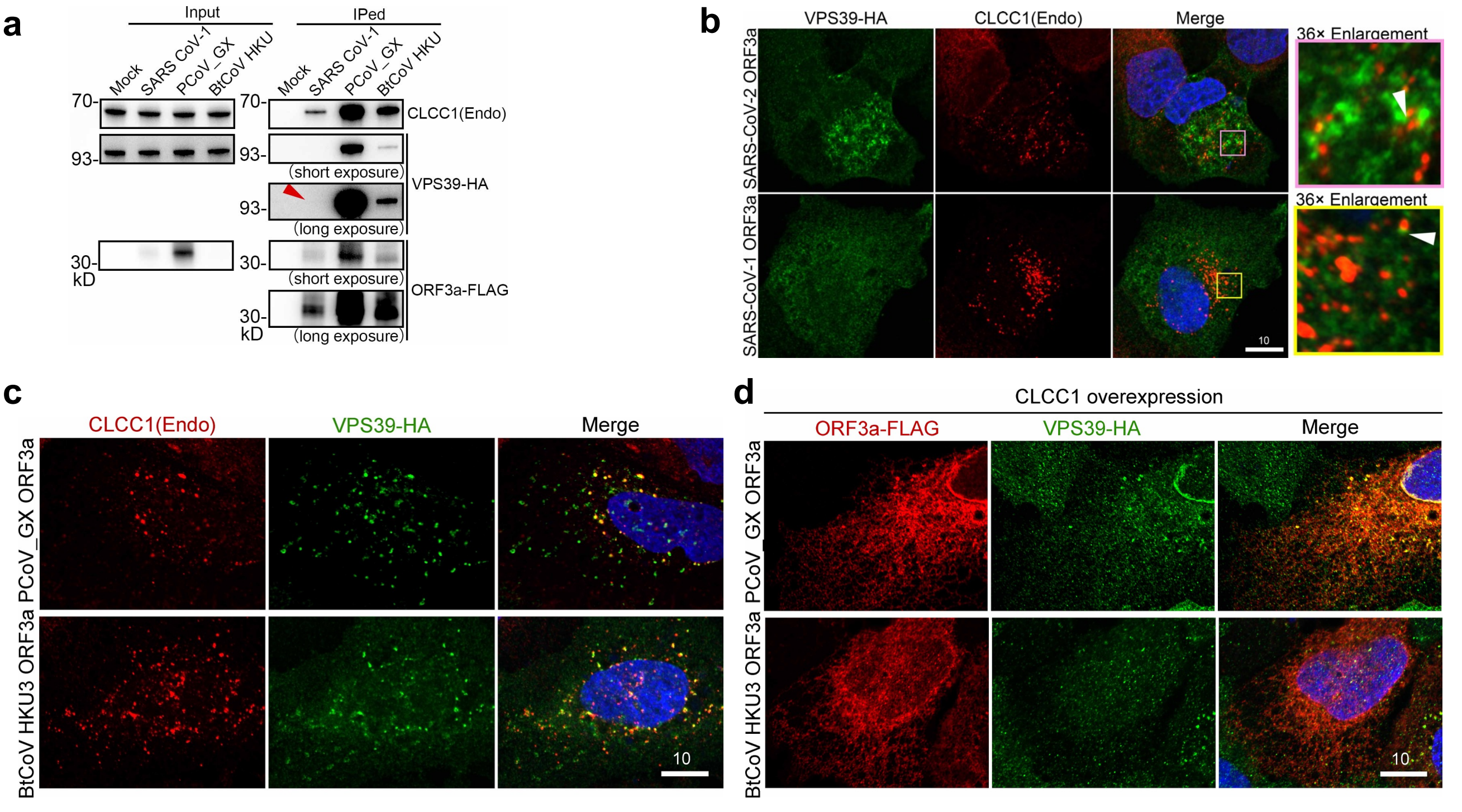

Supplementary Fig. S8 Function of ORF3a among beta-coronaviruses in interacting with CLCC1.

a, Conservation analysis of ORF3a interactions with VPS39 and CLCC1 across beta-coronaviruses. FLAG-tagged ORF3a homologs were transiently expressed in 293FT cells stably expressing HA-VPS39, followed by immunoprecipitation using anti-FLAG M2 magnetic beads. Co-precipitated proteins were analyzed by immunoblotting with antibodies targeting CLCC1, FLAG (ORF3a), and HA (VPS39). Long and short exposures were provided for the same blot (a). Red arrow denotes absence of HA signal in precipitates.

b-c, Subcellular localization analysis of VPS39 and endogenous CLCC1 in ORF3a-expressing U2OS cells. HA-VPS39 and CC1 were detected using anti-HA and anti-CLCC1 antibodies, respectively. White arrows indicate the gap between CLCC1 puncta and VPS39 signals.

d, Immunofluorescence analysis of VPS39 and FLAG-tagged ORF3a in the U2OS cells over-expressing CLCC1. VPS39 and ORF3a were detected by the antibodies against HA and FLAG, respectively.

Scale bar in **b-d**, 10 μm.

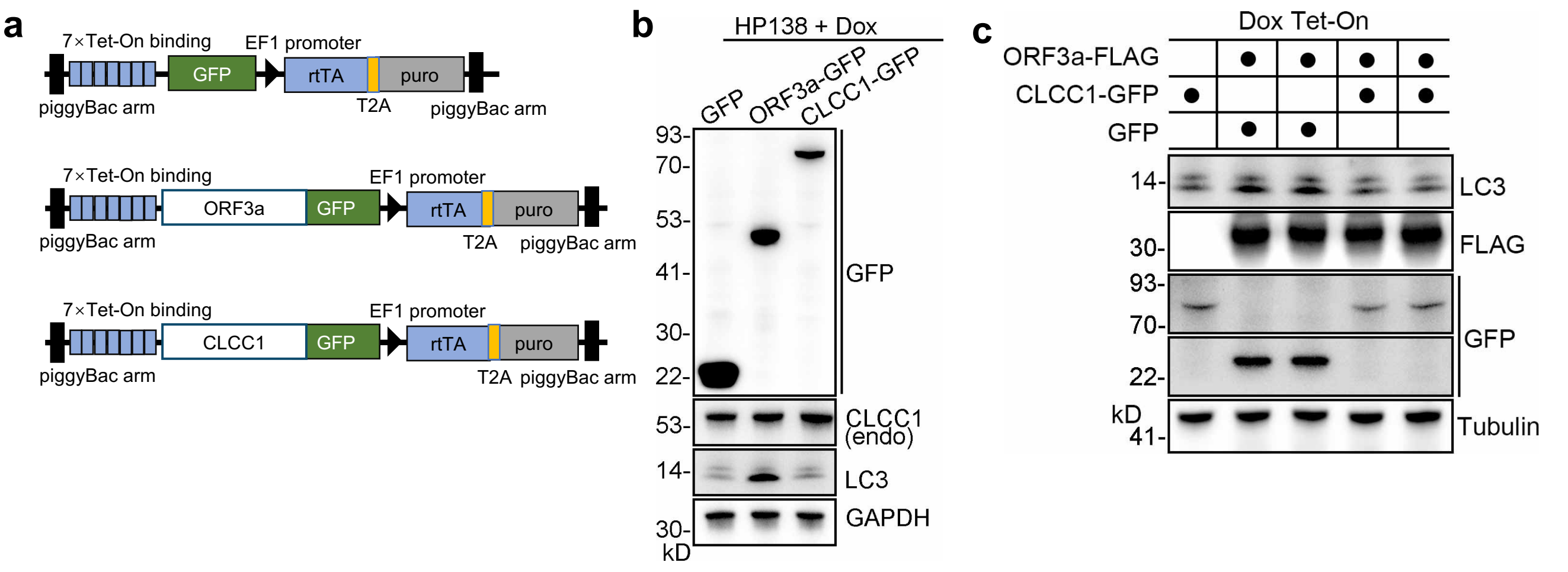

Supplementary Fig. S9 Inducible expression of SARS-CoV-2 ORF3a-GFP but not GFP and CLCC1-GFP enhances LC3 lipidation.

a. Illustration of the inducible expression system for GFP, SARS-CoV-2 ORF3a-GFP, and CLCC1-GFP.

b, The expression of SARS-CoV-2 ORF3a-GFP, but not GFP and CLCC1-GFP, promotes LC3 lipidation. 293FT stably expressing GFP, GFP-tagged SARS-CoV-2 ORF3a, or GFP-tagged CLCC1 were treated with Dox (1 μg/ml) to induce expression.

c, Increased CLCC1 ameliorates LC3 lipidation triggered by SARS-CoV-2 ORF3a. Flag-tagged SARS-CoV-2 ORF3a was transiently expressed in the inducible cell line expressing CLCC1-GFP. After 6 hours of transfection, doxycycline (Dox, 1 μg/ml) was added to induce CLCC1 expression. The inducible cell line expressing GFP served as a negative control.

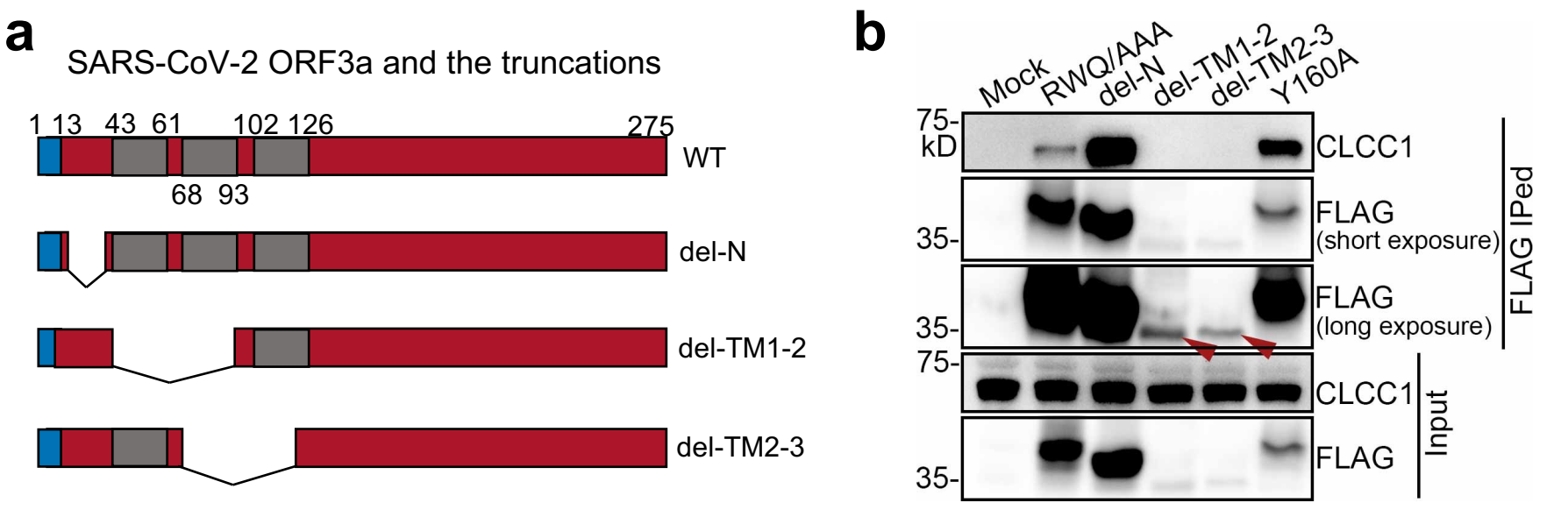

Supplementary Fig. S10 TM2 of SARS-CoV-2 ORF3a may be required for its binding to CLCC1.

a, Schematic illustration of the truncation mutants of SARS-CoV-2 ORF3a. The del-N deletes most of the soluble N-terminus, removing residues Ile20 to Ser40. The del-TM1-2 deletes both TM1 and TM2, removing residues Gly49 to Val97. The del-TM2-3 deletes TM2 and TM3, removing residues Trp68 to Ile124.

b, Domain mapping of SARS-CoV-2 ORF3a-CLCC1 interaction. Truncated FLAG-tagged ORF3a variants were transiently expressed in 293FT cells, immunoprecipitated with anti-FLAG beads, and analyzed by immunoblotting with anti-CLCC1 and anti-FLAG antibodies.

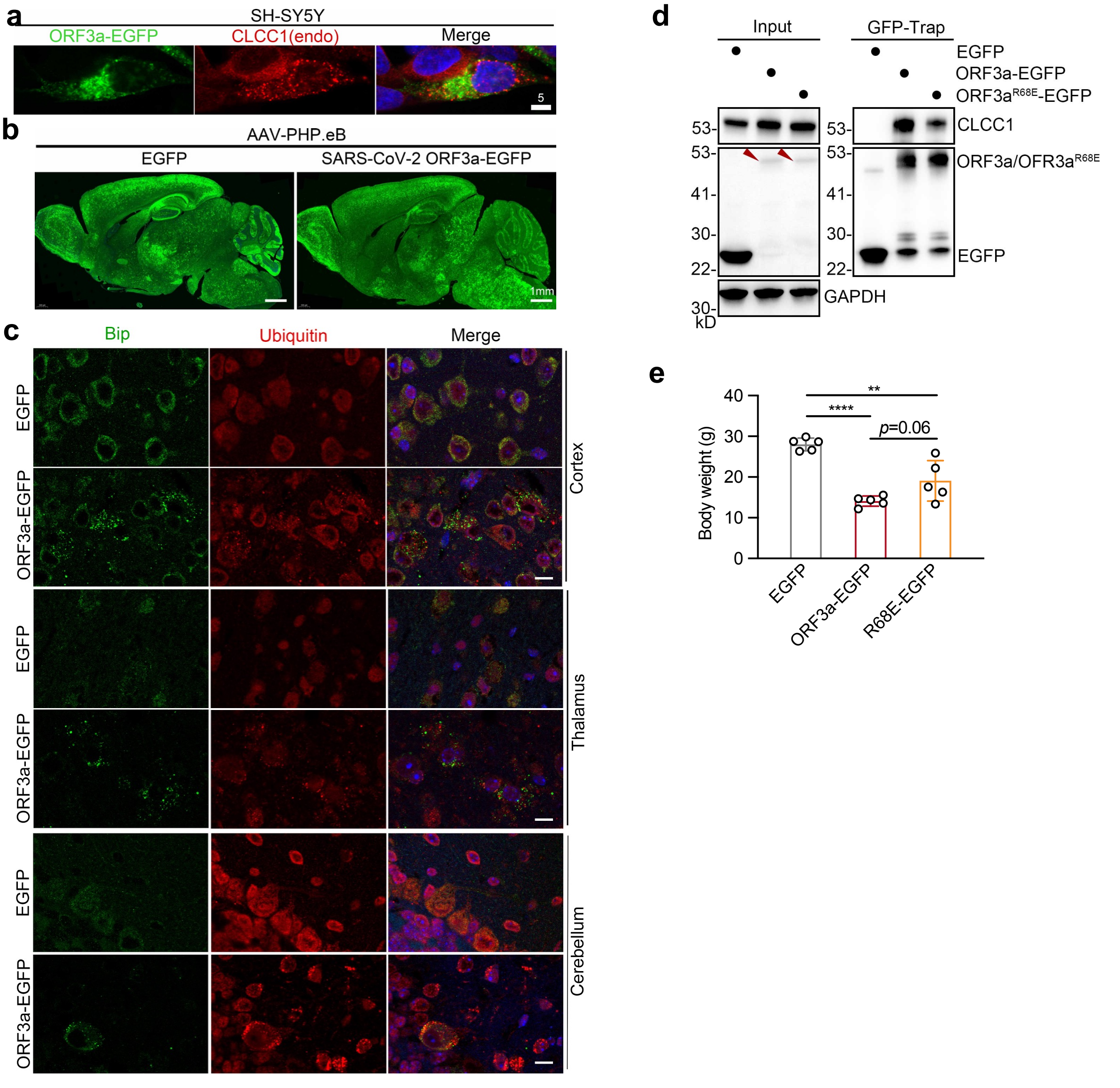

Supplementary Fig. S11

Supplementary Fig. S11| SARS-CoV-2 ORF3a causes ER stress and ubiquitinated protein accumulation.

a, Immunofluorescence analysis of endogenous CLCC1 in SH-SHY5Y cells expressing ORF3a-GFP.

b, EGFP and SARS-CoV-2 ORF3a-EGFP expression in mouse brains. The mice were sacrificed 20 days post AAV-PHP.eB administration. The expression of EGFP and SARS-CoV-2 ORF3a-EGFP was documented by GFP staining. Note that the contrast of SARS-CoV-2 ORF3a-EGFP was adjusted to a similar extent to the EGFP group because of the low expression level of SARS-CoV-2 ORF3a-EGFP in the brain.

c, SARS-CoV-2 ORF3a expression in mouse brains triggers Bip upregulation and ubiquitinated protein accumulation in the cortex, thalamus, and cerebellum.

d, R68E mutation weakens the interaction ORF3a with endogenous CLCC1. The brain lysates were subjected to GFP-Trap immunoprecipitation followed by immunoblot analysis (**f**). GFP-trap beads captured the EGFP-tagged SARS-CoV-2 ORF3a, ORF3a^{R68E}, and EGFP. Red arrows identify EGFP-tagged SARS-CoV-2 ORF3a and ORF3a^{R68E} in input.

e, SARS-CoV-2 ORF3a^{R68E} expression leads to weight loss in mice.

Scale bar, 5 μm in **a** and **c**, 1 mm in **b**. Values are presented as mean ± SD. **P < 0.01, ***P < 0.001 by Student's *t*-test (**e**).

Supplementary Table S1. Differentially Expressed Genes (DEGs) between 293FT cells expressing SARS-CoV-2 ORF3a and GFP; Associated with Fig. 2.

Supplementary Table S2. KEGG pathway analysis of the up-regulated and down-regulated DEGs in 293FT cells expressing SARS-CoV-2 ORF3a; Associated with Fig. 2.

Supplementary Video S1 3D-reconstruction of 293FT cell expressing APEX2-tagged SARS-CoV-2 ORF3a .

DAB-treated cells were processed for serial TEM sectioning and tomographic reconstruction. The video demonstrates preferential localization of ORF3a (dark DAB deposits) at plasma membranes, ER membranes, nuclear envelopes, lysosomal compartments, and autophagosomal structures.

Supplementary Video S2 SARS-CoV-2 ORF3a expression leads to weight loss and movement disability in mice.

Wild-type mice received injections of AAV containing SARS-CoV-2 ORF3a-EGFP or EGFP alone at P22 and were observed at P40. Compared to EGFP-expressing controls, the mice expressing SARS-CoV-2 ORF3a-EGFP exhibit slimness, kyphosis, and movement disability.
